# Automated intracranial vessel segmentation of 4D flow MRI data in patients with atherosclerotic stenosis using a convolutional neural network

**DOI:** 10.3389/fradi.2024.1385424

**Published:** 2024-06-04

**Authors:** Patrick Winter, Haben Berhane, Jackson E. Moore, Maria Aristova, Teresa Reichl, Julian Wollenberg, Adam Richter, Kelly B. Jarvis, Abhinav Patel, Fan Z. Caprio, Ramez N. Abdalla, Sameer A. Ansari, Michael Markl, Susanne Schnell

**Affiliations:** ^1^ Department of Medical Physics, Faculty of Mathematics and Natural Sciences, University of Greifswald, Greifswald, Germany; ^2^ Department of Radiology, Northwestern University, Feinberg School of Medicine, Chicago, IL, United States; ^3^ Department of Neurology, Northwestern University, Feinberg School of Medicine, Chicacgo, IL, United States; ^4^ Department of Experimental Physics V, University of Wuerzburg, Wuerzburg, Germany; ^5^ Department of Diagnostic Radiology, University Hospital of Greifswald, Greifswald, Germany

**Keywords:** atherosclerosis, intracranial, segmentation, deep learning, stenoses, 4D flow, stenosis, convolutional neural network

## Abstract

**Introduction:**

Intracranial 4D flow MRI enables quantitative assessment of hemodynamics in patients with intracranial atherosclerotic disease (ICAD). However, quantitative assessments are still challenging due to the time-consuming vessel segmentation, especially in the presence of stenoses, which can often result in user variability. To improve the reproducibility and robustness as well as to accelerate data analysis, we developed an accurate, fully automated segmentation for stenosed intracranial vessels using deep learning.

**Methods:**

154 dual-VENC 4D flow MRI scans (68 ICAD patients with stenosis, 86 healthy controls) were retrospectively selected. Manual segmentations were used as ground truth for training. For automated segmentation, deep learning was performed using a 3D U-Net. 20 randomly selected cases (10 controls, 10 patients) were separated and solely used for testing. Cross-sectional areas and flow parameters were determined in the Circle of Willis (CoW) and the sinuses. Furthermore, the flow conservation error was calculated. For statistical comparisons, Dice scores (DS), Hausdorff distance (HD), average symmetrical surface distance (ASSD), Bland-Altman analyses, and interclass correlations were computed using the manual segmentations from two independent observers as reference. Finally, three stenosis cases were analyzed in more detail by comparing the 4D flow-based segmentations with segmentations from black blood vessel wall imaging (VWI).

**Results:**

Training of the network took approximately 10 h and the average automated segmentation time was 2.2 ± 1.0 s. No significant differences in segmentation performance relative to two independent observers were observed. For the controls, mean DS was 0.85 ± 0.03 for the CoW and 0.86 ± 0.06 for the sinuses. Mean HD was 7.2 ± 1.5 mm (CoW) and 6.6 ± 3.7 mm (sinuses). Mean ASSD was 0.15 ± 0.04 mm (CoW) and 0.22 ± 0.17 mm (sinuses). For the patients, the mean DS was 0.85 ± 0.04 (CoW) and 0.82 ± 0.07 (sinuses), the HD was 8.4 ± 3.1 mm (CoW) and 5.7 ± 1.9 mm (sinuses) and the mean ASSD was 0.22 ± 0.10 mm (CoW) and 0.22 ± 0.11 mm (sinuses). Small bias and limits of agreement were observed in both cohorts for the flow parameters. The assessment of the cross-sectional lumen areas in stenosed vessels revealed very good agreement (ICC: 0.93) with the VWI segmentation but a consistent overestimation (bias ± LOA: 28.1 ± 13.9%).

**Discussion:**

Deep learning was successfully applied for fully automated segmentation of stenosed intracranial vasculatures using 4D flow MRI data. The statistical analysis of segmentation and flow metrics demonstrated very good agreement between the CNN and manual segmentation and good performance in stenosed vessels. To further improve the performance and generalization, more ICAD segmentations as well as other intracranial vascular pathologies will be considered in the future.

## 1 Introduction

Intracranial 4D flow magnetic resonance imaging (MRI) is a promising imaging modality enabling 3D visualization and quantification of blood flow values (1), and flow-related parameters (2). Previous studies already demonstrated that this phase-contrast (PC) technique can be successfully applied to a variety of pathologies, for example, to explore the hemodynamic alterations due to aneurysms (3), cerebral arteriovenous malformations (4) and intracranial atherosclerotic disease (ICAD) (5). The quantitative analysis of intracranial 4D flow MRI, however, still poses several practical challenges due to its complexity and time-consuming manual 3D segmentation required for quantification, especially in the presence of pathologies. Besides the complicated intracranial vessel geometry, structural and morphological changes due to atherosclerotic plaque formation can aggravate manual segmentation, thus leading to low reproducibility. For example, vascular stenoses can lead to flow artifacts and signal loss, hampering an accurate segmentation of the vessel. To improve the accuracy, reproducibility, and robustness of the analysis of hemodynamic parameters and to accelerate data analysis, an accurate, automated segmentation algorithm for stenosed intracranial vessels is required. A large variety of techniques have been developed to address the problem of semi or fully-automatic vessel segmentation (6). While previous segmentation approaches already drastically improve temporal efficiency in comparison to manual segmentations, they still often require manual labor and lack robustness and consistency, therefore often requiring user interactions from the technologist (7).

With the recent rise of deep learning and convolutional neural networks (CNN), new algorithms have been proposed, promising more reliable and less user-dependent vascular segmentation (8). In particular, the introduction of the U-NET and its variants (9, 10) led to a broad range of new techniques for vessel segmentation using clinical vessel imaging techniques, already achieving very good agreement in comparison to manual segmentations performed by radiologists (7, 11). For example, U-NET was successfully applied for segmentations of cerebral vessels in time-of-flight (TOF) magnetic resonance angiography (MRA) images in patients with cerebrovascular disease (12) and for digital subtraction angiography (DSA) images in patients with intracranial aneurysms (13).

However, up to now, most deep learning-based segmentation approaches for intracranial vessels use TOF or contrast-enhanced (CE) MRA images, as well as DSA, or computed tomography (CT) angiography images while there are no approaches based on 4D flow MRI to the knowledge of the authors at the time of writing this manuscript. For intracranial 4D flow MRI applications, non-deep learning algorithms such as centerline processing schemes (5, 14) or using the standard difference of mean velocity (15) were proposed. The use of 4D flow MRI for deep-learning-based segmentations, however, would have several advantages in comparison to other imaging and segmentation modalities: First, no registration is required to spatially match the segmentation of a different imaging modality with the 4D flow MRI measurement, which can be computationally expensive and prone to errors. Secondly, techniques such as dual-VENC-4D flow MRI enable the assessment of morphological and functional information of the complete vascular tree including both the arteries and the veins in a single measurement (1) while with TOF usually, two separate measurements (angio- and venograms) are necessary. Finally, the use of deep learning has the potential to reduce the aforementioned dependency on user interactions from the technologist.

Recently, Berhane et al. proposed a U-NET-based convolutional neural network technique for the automated segmentation of the aorta using phase-contrast angiography (PCMRA) images derived from aortic 4D flow MRI (16). Here, the automated segmentation achieved an excellent agreement with manual segmentations, however, its use for intracranial 4D flow MRI and its performance in pathology such as intracranial atherosclerotic stenosis still needs to be investigated.

Therefore, in this study, the neural network developed for aortic 4D flow measurements (16) was re-trained for the automated vessel segmentation of intracranial 4D flow MRI in healthy controls and intracranial atherosclerotic stenosis patients. To assess possible differences in segmentation performance between cases with and without disease, the results were compared with segmentations of healthy controls. For performance assessment, Dice score, Hausdorff distance, and average symmetrical surface distance were computed using two independent manual observers as reference. In addition, parameters such as peak velocity, flow rate, and flow conservation error were computed and compared to the manual analyses. Furthermore, the segmentation performance in stenosed vessels was analyzed and compared with segmentation results based on black blood vessel wall imaging (VWI).

## 2 Methods

### 2.1 Study cohort

As part of a clinical ICAD protocol at Northwestern Memorial Hospital, 4D flow MRI scans were acquired in ICAD patients. 35 cases expressed severe stenosis with >70% constriction, 25 cases expressed moderate stenosis with >50% and <70% constriction, and 5 cases had mild stenosis with <50% constriction. Additional 3 cases didn't have a significant stenosis. The data acquired between 2014 and 2022 were retrospectively selected (*n* = 68, *n* = 30 women) for this institutional review board (IRB) approved study. All ICAD-related stenoses were confirmed using the clinical electronic medical record, MRI/MRA, and MR vessel wall imaging review by two interventional neuroradiologists (RA, SAA).

In addition, 4D flow MRI data of healthy volunteers (*n* = 86, *n* = 43 women) was included in this study. Informed consent was obtained from all volunteers. An overview of all patients and volunteers can be found in Table 1 (White background: all cases. Shaded background: Testing cohorts only).

**Table 1:** Sex, median age, BMI, and heart rate (max and min values in brackets) for the control and ICAD group, respectively (top: all cases. Bottom: testing cases).

Group	*N*	Age (years)	BMI (kg/m^2^)	Heart rate (bpm)
Controls (all)	**86** (43 female)	**55** (19–76)	**27.3** (19.4–49.1)	**78.1** (50.0–121.1)
ICAD (all)	**68** (30 female)	**64** (34–85)***	**28.0** (19.9–41.3)	**86.4** (69.1–138.1)**
Controls (test)	**10** (7 female)	**30** (23–76)	**26.5** (19.4–38.7)	**90.8** (66.0–121.1)
ICAD (test)	**10** (6 female)	**64** (36–76)^†^	**29.7** (21.9–35.9)	**86.4** (69.1–138.2)

The heart rate was determined with HR=1/period, using the estimation $\mathrm{period}=(N_{\mathrm{CardiacPhases}}+1)\cdot \mathrm{TR}$ for the cardiac periods. Statistical significance between controls and ICAD (all cases).

All results are stated as median values (in bold) and range values (in brackets).

* *p* < 0.05.

** *p* < 0.01.

*** *p* < 0.001. Statistical significance between controls and ICAD (test cases).

^†^ *p* < 0.05.

### 2.2 MRI

Patients were scanned using a clinical MRI protocol including 4D flow MRI and VWI with 3D-T_1_-SPACE (17) (sampling perfection with application-optimized contrasts using different flip angle evolutions). In addition, 4D flow MRI scans were acquired in healthy volunteers. The VWI parameters were: Spatial resolution 0.52 mm × 0.52 mm × 0.70 mm, TR = 800 ms, TE = 23 ms. The relevant scan parameters for the 4D flow scans can be found in Table 2. For both cohorts kt-GRAPPA accelerated (*R* = 5) dual-VENC 4D Flow MRI was utilized (1). The field of view (FOV) was positioned to cover the circle of Willis (CoW), including the basilar artery (BA), left and right internal carotid arteries (ICA), middle cerebral arteries (MCA), anterior cerebral arteries (ACA), the posterior cerebral arteries (PCA), the posterior communicating arteries (PCOM), the superior cerebral arteries (SCA), the vertebral arteries (VA) as well as the superior sagittal sinus (SSS), straight sinus (STR) and left and right transverse sinus (TS). All measurements were performed at 3 T using a Prisma Fit or Skyra (both Siemens Healthineers Inc., Erlangen, Germany).

**Table 2:** Scan parameters for dual-VENC 4D flow MRI.

Parameters\group	Controls	ICAD
TR [ms]	82.6–85.4	42.0–86–8
TE [ms]	3.25–3.52	3.25–3.41
Voxel size [mm]	0.98	0.98–1.11
Slice thickness [mm]	1.0	1.0–1.2
Number of slices (N_Slices_)	40–44	26–60
Number of cardiac phases (N_CardiacPhases_)	5–11	5–18
Flip angle [°]	15	15
Low venc/high venc [m/s]	0.5-0.6/1.0–1.2	0.5–0.6/1.0–1.2
MRI system	Prisma Fit	Skyra

### 2.3 Post-processing

A custom-built MATLAB (The MathWorks, Natick, USA) tool was used for Eddy current correction, noise masking, and anti-aliasing of the phase difference images (1, 18). Phase-contrast MRA images (PCMRA) were calculated using the pseudo-complex difference method (Equations 1, 2) (1, 3):

(1) $$\mathrm{PCMRA}=\{\begin{array}{c} \frac{1}{N}\sum_{i=1}^{N}I_{i}^{Mag}\cdot \sin\left(\frac{\pi \cdot v_{i}}{V_{ENC}}\right)\cdot v_{i},\;\\ \frac{1}{N}\sum_{i=1}^{N}I_{i}^{Mag},\; \end{array}\underset{\mathrm{otherwise}}{v_{i}<\frac{2}{3}V_{ENC}}$$

with

(2) $$v_{i}=\sqrt{v_{x,i}^{2}+v_{y,i}^{2}+v_{z,i}^{2}}$$

Here, $I_{i}^{Mag}$ denotes the magnitude images derived from the 4D flow measurement, *i* the index and *N* the total number of cardiac phases (1, 3).

For the creation of training and validation data, the PCMRA images were manually segmented in MIMICS (Mimics, Materialise, Belgium). This was achieved by applying a threshold to remove noisy voxels. Subsequently, the neurovascular architecture was identified using the region-growing tool in MIMICS to select areas of intracranial vessel voxels. Noisy voxels captured with the region-growing processes were manually removed from the segmentation. All cases were subsequently edited by a second investigator of more than 10 years of experience (PW). This second step was to achieve consensus segmentations so that these Observer 1 segmentations can serve as “ground truth”. Due to the lack of availability of the commercial software MIMICS, the second user changed to the open-source software 3DSlicer (Slicer 5.2.2., SlicerCommunity) ensuring repeatability. All initial segmentations were performed by operators with at least 2 years of experience (MA, JM, AR).

### 2.4 Automated segmentation using convolutional neural networks

**Figure 1:**
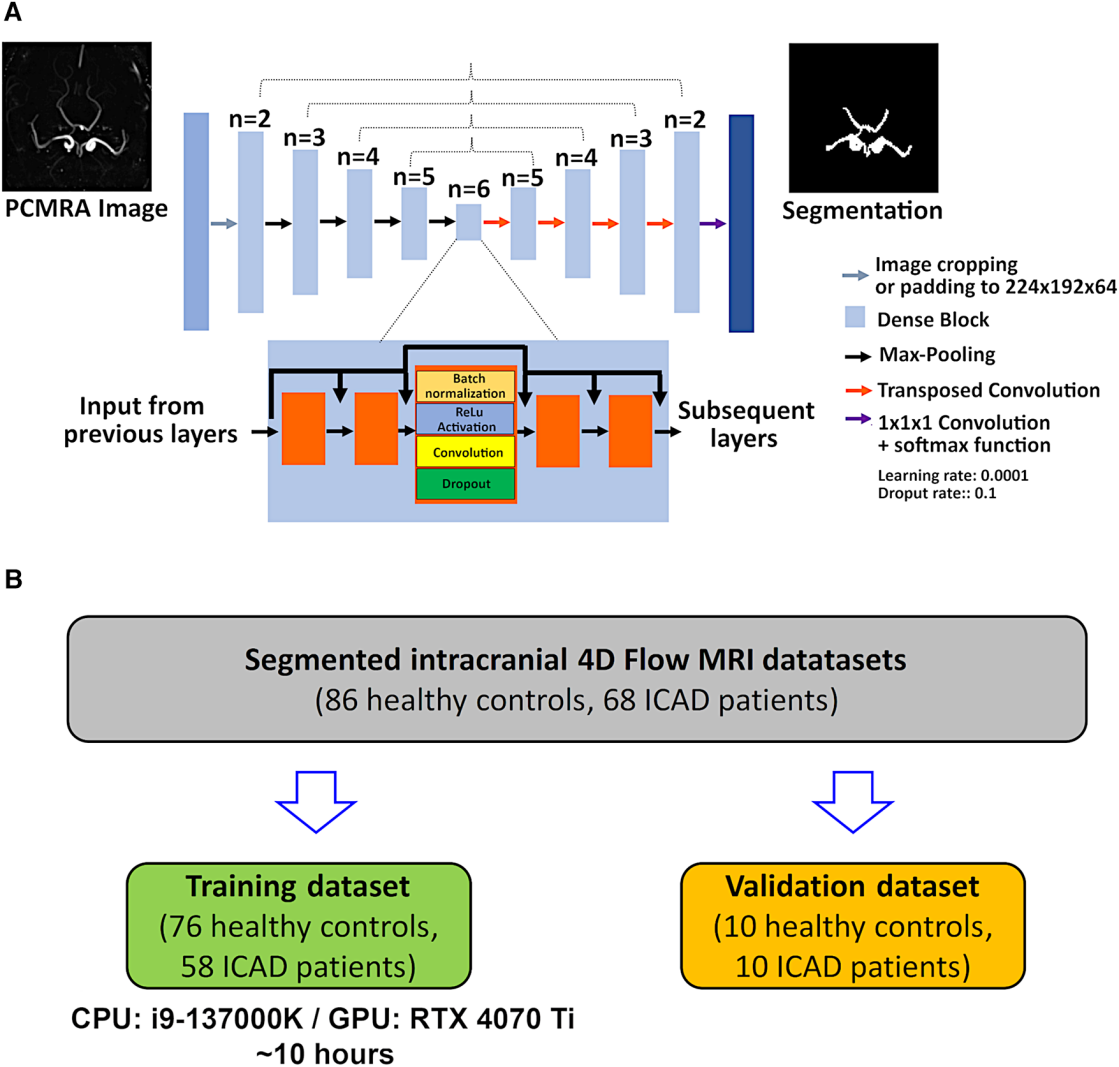
(**A**) Layer structures of the CNN. A symmetrical design is used based on the 3D U-Net architecture. Different from the original approach, dense blocks are implemented into each layer. Dense blocks enable the regulation of the growth of the CNN while efficiently applying feature maps extracted through the CNN by using concatenation after each convolution layer. Dense blocks consist of the serial application of batch normalization, activation with a linear rectifier unit (ReLu) a 3 × 3 × 3 convolution, and a dropout layer (dropout rate 0.1). (**B**) Chart illustrating the splitting of the patient and control data into the training and validation datasets.

For the automated segmentation, the CNN developed by Berhane et al. was used (16). The network consists of a 3D U-Net [(10), see Figure 1A]. The original convolution layers were replaced by dense blocks (19), as described previously (16). For each dense block, batch normalization, a linear rectifier unit (ReLu), a 3D convolution (3 × 3 × 3), and a dropout layer (dropout rate 0.1) were computed. For the training, the calculated PCMRA images [see Equation (1)] were center-cropped or padded to obtain a fixed dimension of 224 × 192 × 64 and used as input for the CNN. No patching of the data was used. Instead, center-cropping was applied to reduce dimensions, since the intracranial vessels are always located around the center. Furthermore, no data augmentation was performed since the intracranial 4D flow scans are always acquired in the same orientation. To increase efficiency, all prior feature maps were concatenated and used as inputs for the subsequent layers (16). In the encoding part of the U-Net (see left-hand side of Figure 1A), a max-pooling layer is applied for downsampling while transposed convolution is used for upsampling in the decoding part (see right-hand side of Figure 1A). In the final layer, a 1 × 1 × 1 convolution and a softmax function are applied. The last step generates a binary value for each voxel of the input image (0: background, 1: foreground). Segmentation masks were created by selecting the class with the highest probability per voxel. For the training, a composite loss function (softmax-cross entropy and Dice loss), a batch size of 1, a learning rate of 0.0001, and 300 epochs were used. All computations were performed in *Python 3.6.13* (Python Software Foundation, Beaverton, OR) with *Tensorflow* 2.4.0 on a 13th Gen Intel Core i7-13700 (2,100 MHz, 16 Cores) CPU with an NVIDIA GeForce RTX 4070 Ti GPU with 16 GB VRAM. A cohort of 134 randomly selected cases (76 controls, 58 ICAD patients) were used for the training while the remaining 20 cases (10 controls, 10 ICAD patients: four with severe, four with moderate, one with mild, one without significant stenosis. See Table 1 for group statistics) were used for testing (see Figure 1B).

### 2.5 CNN performance analysis

To compare the performance between the manual and automated segmentation, the Dice score (DS), Hausdorff distance (HD), and average symmetrical surface distance (ASSD) were calculated using (Equations 3–5) (20):

(3) $$\mathrm{DS}(X,Y)=\frac{2*|X\cap Y|}{\left|X|+|Y\right|}$$

(4) $$\mathrm{HD}(X,Y)=\max\left(\underset{y\in Y}{\max}\left(\underset{x\in X}{\min}d(x,y)\right),\;\underset{x\in X}{\max}\left(\underset{x\in X}{\min}d(x,y)\right)\right)$$

(5) $$\mathrm{ASSD}(X,Y)=\left(\frac{1}{|X|+|Y|}\right)\left(\sum_{x=1}^{X}\underset{y\in Y}{\min}d(x,y)+\sum_{y=1}^{Y}\underset{x\in Y}{\min}d(x,y)\right)$$

Here, *X* and *Y* are binary segmentation masks and *d* is the Euclidian distance between both segmentations. For the segmentation analysis, DS, HD, and ASSD were computed for:

(a) CoW and sinuses.
(b) Only the CoW.
(c) Only the sinuses.

The calculation of segmentation metrics was performed in MATLAB (DS) and Python (HD and ASSD). For visual presentations of the segmentation masks, Ensight 10.02 (CEI, inc., USA) was used.

### 2.6 Flow analysis

Magnitude, velocity, and segmentation data were imported into a semi-automatic MATLAB analysis tool (5). First, centerlines were created automatically and perpendicular analysis planes were placed equidistantly along the vessels at a 0.25 mm distance. Subsequently, lumen cross-sectional areas, peak velocity, and flow rates were extracted for all analysis planes. Planes close to branches and bifurcations were excluded to avoid systematic errors in the flow estimation. The same analysis planes were used for both the manual and the automated segmentations. For statistical comparisons between manual and automated segmentations, the median cross-sectional area, peak velocity, and temporally averaged flow rate values were calculated over all analysis planes for each vessel of interest. The vascular analysis was subdivided into:

(a) large arteries (BA, ICA, MCA),
(b) small arteries (ACA, PCA, PCOM, SCA, VA),
(c) sinuses (TS, STR, SSS).

In addition, the internal consistency of the flow rate was assessed by determining the flow conservation error (fce) for the arteries (Equation 6) (4):

(6) $$\mathrm{fce}=\left\|1-\frac{total\;flow\;in\;ACAs,\;MCAs,\;PCOMs}{total\;flow\;in\;ICAs}\right\|$$

### 2.7 Performance analysis in stenosed vessels

For an analysis of the segmentation performance in stenosed vessels, one ICAD patient with moderate stenosis (>50% constriction in the right MCA, male, 80 years old) and two patients with severe stenosis (>70% constriction in the right MCA, female, 68 years old, >70% constriction in the right ICA, female, 61 years old) were additionally validated with segmentations obtained from black blood VWI.

For comparison with VWI, rigid registration was applied using the SPM12 MATLAB tool box (21) to align the 3D-T_1_-SPACE images with the 4D flow images. Subsequently, 3D volume analysis of the black blood images was performed using a home-built 3D framework (22, 23). Based on an interactive specialized Dijkstra algorithm (24), the centerline, vessel volume and vessel wall were extracted and visualized. Beforehand, the black blood images were processed in multiple steps: First, a prior median filter (3×3×3) was applied to reduce noise and to enhance the contrast for the Dijkstra-searching algorithm, which was introduced by manually set seed points. In the next step, the volume of the stenosed vessel was extracted along the centerline. In addition, a vertex model of the desired vessel structure was generated using a Marching Cube algorithm (25). Subsequently, the volume was imported to MATLAB. Since the SPM12 co-registration was not perfect and a few voxels off, a second rigid registration using the FLIRT (flexible image reconstruction toolbox) MATLAB tool box (26) was applied to co-register the VWI segmentation with the 4D flow segmentations.

Using the VWI segmentation as reference, DS, HD and ASSD were calculated for both manual segmentations and the automated segmentation. In addition, the lumen cross-sectional area profiles determined with the 4D flow segmentations were compared with segmentations obtained from the T_1_-SPACE images. Within a region of interest around the stenosis, the time-resolved median flow rates were determined for the automated and the Observer 1 and Observer 2 segmentations, respectively.

### 2.8 Interobserver study

For interobserver comparisons of standard manual segmentations, all datasets from the testing cohort (10 controls, 10 ICAD) were segmented by an additional observer (Observer 2) with medical background and one year of experience with 4D flow MRI, who was not part of the original segmentation instance (JW). The second observer was blinded to the original segmentations of the testing cases. All segmentations were performed in 3DSlicer and the segmentations were verified by a second investigator (PW) to avoid systematic errors with the segmentation process. Using the original manual (Observer 1) and the automated segmentation as a reference, DS, HD, and ASSD were computed and compared with the results from the automated segmentation vs. Observer 1 analysis. In addition, flow parameters and cross-sectional areas were compared using the same analysis planes as for the CNN segmentation and the segmentation performance was analyzed in the three stenosis cases described in section 2.7.

### 2.9 Statistical analysis

Cross-sectional areas, as well as flow metrics (peak velocity, flow rate, fce) derived from the automated segmentation and the second observer, were compared with results from the Observer 1 segmentation using correlation and Bland-Altman analysis. Using the Observer 1 segmentation as a reference, interclass correlation coefficients (ICC), relative bias, and limit of agreement (LOA) were assessed for all parameters. Normality was tested using a Shapiro–Wilk test. Depending on normality, a Mann–Whitney *U*-test or an unpaired *t*-test was utilized for statistical evaluations. A *p*-value <0.05 was considered statistically significant. All statistical analyses were performed in MATLAB.

## 3 Results

### 3.1 Performance of the automated segmentation

**Figure 2:**
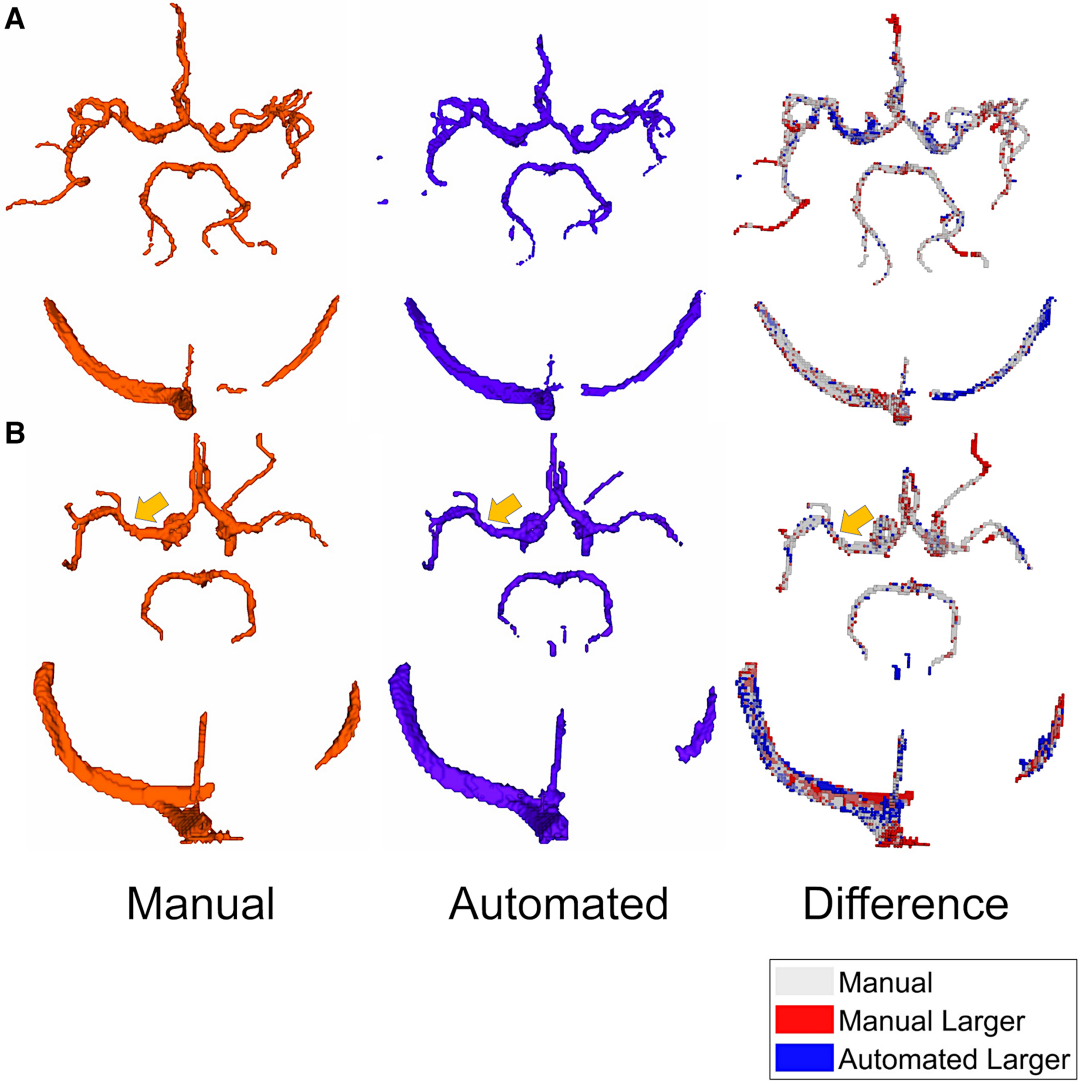
Exemplary results for manual (red) and CNN (blue) segmentations of a control case (**A**) DS = 0.89, HD = 5.09 mm, ASSD = 0.11 mm) and an ICAD case (**B**) DS = 0.84, HD = 3.77 mm, ASSD = 0.15 mm). Difference maps on the right indicate regions of over- (blue) and underestimation (red) of the automated segmentation. Orange arrows mark the location of a severe stenosis in the left MCA.

The training took approximately 10 h and the time required for a single CNN segmentation was 2.2 ± 1.0 s. Figure 2 displays exemplary results for Observer 1 (red) and CNN (blue) segmentations of a control case (Figure 2A) and an ICAD case (Figure 2B). Difference maps indicate regions of over- (blue) and underestimation (red) of the automated segmentation.

Table 3 displays the median values and the range of the DS, HD and ASSD for a comparison between the CNN segmentation and the original manual segmentation. No significant differences were observed when comparing the DS and HD values of the control group with the ICAD group (*p* ≥ 0.19). The average DS values were (mean ± STD) for CoW + sinuses: 0.86 ± 0.04 (controls) and 0.83 ± 0.04 (ICAD, *p* = 0.31). For CoW only: 0.85 ± 0.03 (controls) and 0.85 ± 0.04 (ICAD, *p* = 0.81). For sinuses only: 0.86 ± 0.06 (controls) and 0.82 ± 0.07 (ICAD, *p* = 0.19). Average HD values were for CoW + sinuses: 7.8 ± 1.6 mm (controls) and 7.9 ± 1.9 mm (ICAD, *p* = 0.89). For CoW only: 7.2 ± 1.5 mm (controls) and 8.4 ± 3.1 mm (ICAD, *p* = 0.28). For sinuses only: 6.6 ± 3.7 mm (controls) and 5.7 ± 1.9 mm (ICAD, *p* = 0.65).

**Table 3:** Performance results for the automated segmentation framework.

Parameter/location	Automated vs. Observer 1	
DS	Controls	ICAD
CoW + sinus	**0.87** (0.78–0.92)	**0.85** (0.70–0.87)
CoW	**0.85** (0.79–0.92)	**0.85** (0.81–0.91)
Sinus	**0.87** (0.74–0.94)	**0.84** (0.65–0.87)
HD [mm]	Controls	ICAD
CoW + sinus	**7.6** (5.1–10.3)	**8.4** (3.8–10.4)
CoW	**7.0** (4.4–9.5)	**8.3** (3.1–15.2)
Sinus	**6.3** (3.0–10.3)	**5.9** (3.2–9.5)
ASSD [mm]	Controls	ICAD
CoW + sinus	**0.12** (0.08–0.23)	**0.21** (0.14–0.39)*
CoW	**0.13** (0.08–0.24)	**0.21** (0.06–0.35)*
Sinus	**0.15** (0.05–0.28)	**0.21** (0.13–0.51)

For calculation of DS, HD and ASSD, always the original manual segmentation was used as reference. Analysis of the statistical significance between the control and ICAD group: *p < 0.05. All results are stated as median values (in bold) and range values (in brackets).

For the ASSD values, significant differences between the control and ICAD group were observed for the CoW + sinus segmentation (0.14 ± 0.05 mm vs. 0.21 ± 0.08 mm, *p* = 0.02) and the CoW-only segmentation (0.14 ± 0.04 mm vs. 0.22 ± 0.10 mm, *p* < 0.05). However, for the sinuses-only segmentation, average ASSD values were 0.17 ± 0.08 mm (controls) and 0.22 ± 0.11 mm (ICAD, *p* = 0.23).

### 3.2 Flow metrics

Cross-sectional areas, peak velocity values, and flow rates were determined in the small and large arteries and the sinuses for both cohorts, respectively. Tables 4–6 display the median, range, relative bias, and limits of agreement of all of the above parameters for the automated segmentation vs. Observer 1 and the Observer 1 vs. Observer 2 comparisons. Furthermore, the *p*-values are shown for the Observer 1 vs. automated segmentations, the Observer 1 vs. Observer 2, and the automated segmentation vs. Observer 2 comparison. The top half of each table shows the results for the control group while the bottom half displays the results of the ICAD group. The correlation and Bland-Altman plots can be found in Supplementary Figures S1–S3 in the supplement. No significant differences for all parameters were observed when comparing the Observer 1 with the automated segmentation (*p* ≥ 0.40 for all vessels). The average cross-sectional areas determined with the automated segmentation were (mean ± STD) for the large arteries: 13.3 ± 4.4 mm^2^ (controls) vs. 12.1 ± 4.8 mm^2^ (ICAD, *p* = 0.20). For the small arteries: 5.7 ± 2.1 mm^2^ (controls) vs. 5.3 ± 1.1 mm^2^ (ICAD, *p* = 0.32). For the sinuses: 19.8 ± 11.6 mm^2^ (controls) vs. 22.1 ± 10.0 mm^2^ (ICAD, *p* = 0.48). The average peak velocity values were for the large arteries: 0.61 ± 0.15 m/s (controls) vs. 0.53 ± 0.16 m/s (ICAD, *p* = 0.06). For the small arteries: 0.45 ± 0.14 m/s (controls) vs. 0.41 ± 0.13 m/s (ICAD, *p* = 0.16). For the sinuses: 0.33 ± 0.14 m/s (controls) vs. 0.27 ± 0.12 m/s (ICAD, *p* = 0.15).

**Table 4:** Cross-sectional area values: comparison of the automated and the Observer 2 segmentation with the Observer 1 segmentation.

Controls (*n* = 10)	Large arteries	Small arteries	Sinuses	Segmentation
**Median** (range) [mm^2^]	**11.9** (6.1–20.1)	**5.5** (2.6–9.8)	**15.5** (2.4–41.6)	Observer 1
**Median** (range) [mm^2^]	**12.5** (5.5–23.4)	**5.7** (1.9–10.4)	**17.2** (2.3–42.8)	Automatic
Bias ± LOA [%]	4.6 ± 24.4	2.4 ± 37.8	2.7 ± 35.5	
ICC	0.93	0.86	0.91	
*p*-value (reference: Observer 1)	0.64	0.72	0.79	
**Median** (range) [mm^2^]	**12.5** (5.4–22.8)	**5.3** (1.9–9.7)	**18.3** (4.9–40.7)	Observer 2
Bias ± LOA [%]	4.1 ± 20.1	−1.7 ± 36.3	−2.8 ± 32.7	
ICC	0.96	0.85	0.96	
*p*-value (reference: Observer 1)	0.53	0.80	0.86	
*p*-value (reference: automated)	0.97	0.82	0.83	
ICAD (*n* = 10)	Large arteries	Small arteries	Sinuses	Segmentation
**Median** (range) [mm^2^]	**9.8** (3.9–24.2)	**5.3** (3.1–8.4)	**22.5** (5.9–49.8)	Observer 1
**Median** (range) [mm^2^]	**11.4** (3.3–25.4)	**5.4** (3.4–7.9)	**23.8** (6.9–42.0)	Automatic
Bias ± LOA [%]	4.9 ± 29.1	2.0 ± 31.4	−7.2 ± 46.9	
ICC	0.87	0.71	0.86	
*p*-value (reference: Observer 1)	0.40	0.69	0.56	
**Median** (range) [mm^2^]	**10.8** (4.5–20.8)*	**5.1** (2.0–9.2)	**20.5** (3.8–42.4)	Observer 2
Bias ± LOA [%]	−2.9 ± 36.3	−6.8 ± 53.2	−10.0 ± 54.9	
ICC	0.89	0.57	0.86	
*p*-value (reference: Observer 1)	0.87	0.19	0.47	
*p*-value (reference: automated)	0.25	0.16	0.56	

All results are stated as median values (in bold) and range values (in brackets).

Statistical significance between the control and ICAD group.

* *p* < 0.05.

**Table 5:** Peak velocity values: comparison of the automated and the Observer 2 segmentation with the Observer 1 segmentation.

Controls (*n* = 10)	Large arteries	Small arteries	Sinuses	Segmentation
**Median** (range) [m/s]	**0.56** (0.37–0.91)	**0.43** (0.25–1.0)	**0.28** (0.13–0.67)	Observer 1
**Median** (range) [m/s]	**0.56** (0.40–0.91)	**0.43** (0.25–1.0)	**0.29** (0.12–0.67)	Automatic
Bias ± LOA [%]	0.85 ± 4.0	−0.88 ± 13.5	0.21 ± 1.1	
ICC	>0.99	0.95	>0.99	
*p*-value (reference: Observer 1)	0.82	0.96	0.94	
**Median** (range) [m/s]	**0.55** (0.40–0.91)	**0.43** (0.22–1.0)	**0.28** (0.12–0.63)	Observer 2
Bias ± LOA [%]	0.68 ± 4.3	−2.5 ± 19.7	−0.06 ± 1.1	
ICC	>0.99	0.89	>0.99	
*p*-value (reference: Observer 1)	0.82	0.64	0.94	
*p*-value (reference: automated)	0.99	0.91	0.89	
ICAD (*n* = 10)	Large arteries	Small arteries	Sinuses	Segmentation
**Median** (range) [m/s]	**0.57** (0.27–0.87)	**0.41** (0.23–0.92)	**0.27** (0.08–0.75)	Observer 1
**Median** (range) [m/s]	**0.57** (0.27–0.87)	**0.40** (0.23–0.93)	**0.27** (0.08–0.54)	Automatic
Bias ± LOA [%]	0.04 ± 0.43	−0.03 ± 1.2	−0.01 ± 0.31	
ICC	>0.99	>0.99	>0.99	
*p*-value (reference: Observer 1)	0.98	>0.99	0.67	
**Median** (range) [m/s]	**0.56** (0.27–0.86)	**0.39** (0.23–0.93)	**0.27** (0.08–0.53)	Observer 2
Bias ± LOA [%]	0.33 ± 3.5	0.06 ± 1.09	−0.08 ± 0.75	
ICC	>0.99	>0.99	>0.99	
*p*-value (reference: Observer 1)	0.82	0.97	0.75	
*p*-value (reference: automated)	0.84	0.90	0.90	

All results are stated as median values (in bold) and range values (in brackets).

**Table 6:** Flow rates (automated and Observer 2 vs. Observer 1).

Controls (*n* = 10)	Large arteries	Small arteries	Sinuses	Segmentation
**Median** (range) [ml/s]	**3.5** (1.1–8.8)	**1.2** (0.33–5.0)	**3.0** (0.41–10.3)	Observer 1
Flow conservation error	**0.17** (0.04–0.32)			
**Median** (range) [ml/s]	**3.5** (1.0–9.0)	**1.2** (0.32–4.8)	**2.9** (0.37–10.1)	Automatic
Bias ± LOA [%]	1.8 ± 12.3	4.0 ± 29.5	1.5 ± 17.9	
ICC	>0.99	0.97	>0.99	
*p*-value (reference: Observer 1)	0.92	0.92	0.80	
Flow conservation error	**0.11** (0.04–0.34)			
**Median** (range) [ml/s]	**3.4** (1.2–9.1)	**1.3** (0.27–5.1)	**2.6** (0.64–9.6)	Observer 2
Bias ± LOA [%]	0.99 ± 10.67	0.17 ± 23.5	−2.0 ± 15.0	
ICC	>0.99	0.98	>0.99	
*p*-value (vs. obs. 1/auto)	0.93/0.97	0.83/0.96	0.90/0.92	
Flow conservation error	**0.15** (0.03–0.31)			
ICAD (*n* = 10)	Large arteries	Small arteries	Sinuses	Segmentation
**Median** (range) [ml/s]	**2.4** (0.6–4.8)***	**1.0** (0.4–2.7)*	**3.0** (0.4–6.7)	Observer 1
Flow conservation error	**0.18** (0.01–0.39)			
**Median** (range) [ml/s]	**2.5** (0.6–4.9)***	**1.0** (0.47–2-6)	**2.6** (0.44–6.7)	Automatic
Bias ± LOA [%]	2.1 ± 13.5	2.0 ± 22.8	0.08 ± 18.7	
ICC	0.99	0.97	0.99	
*p*-value (reference: Observer 1)	0.81	0.88	>0.99	
Flow conservation error	**0.18** (0.02–0.38)			
**Median** (range) [ml/s]	**2.4** (0.64–4.87)***	**0.91** (0.29–2.0)	**2.8** (0.25–6.4)	Observer 2
Bias ± LOA [%]	−0.03 ± 12.9	−3.3 ± 28.6	−2.8 ± 18.0	
ICC	0.99	0.95	0.99	
*p*-value (vs. Obs. 1/vs. auto)	0.86/0.67	0.39/0.39	0.71/0.71	
Flow conservation error	**0.16** (0.01–0.46)			

All results are stated as median values (in bold) and range values (in brackets).

Statistical significance between the control and ICAD group.

* *p* < 0.05.

** *p* < 0.01.

*** *p* < 0.001.

For the flow rates, a significant difference was observed between both cohorts in the large arteries: 3.9 ± 1.7 ml/s (controls) vs. 2.7 ± 1.2 ml/s (ICAD, *p* < 0.01). For the small arteries, the mean values were 1.4 ± 0.8 ml/s (controls) vs. 1.0 ± 0.5 ml/s (ICAD, *p* = 0.064). For the sinuses, the flow values were: 3.6 ± 2.6 ml/s (controls) and 3.1 ± 1.9 ml/s (ICAD, *p* = 0.58). When analyzing the Observer 1 segmentation, significant intergroup differences were observed for the flow rates in the large arteries (*p* < 0.01) and small arteries (*p* = 0.044) but not for the sinuses (*p* = 0.67). Using the flow rate values, the flow conservation error was determined for the manual and automated segmentation, respectively. For the control group, the fce was 0.16 ± 0.09 (manual) and 0.15 ± 0.10 (automated). For the ICAD group, the fce was 0.18 ± 0.12 (manual) and 0.20 ± 0.13 (automated). No significant differences were observed between the manual and CNN segmentation (*p* ≥ 0.80 for both groups).

### 3.3 Interobserver comparison

The segmentation time of an individual manual segmentation performed by Observer 2 was 1,103 ± 347 s. On the left of Table 7 the results for the DS, HD, and ASSD for the Observer 2 segmentation values are displayed using the Observer 1 segmentation as a reference. The right side of the table shows the performance analysis of the automated segmentation vs. Observer 2 comparison. No differences were observed when comparing the DS values from the automated segmentation vs. Observer 1 comparison in Table 3 with the Observer 1 vs. Observer 2 comparison (Controls: CoW + sinuses: 0.86 ± 0.03, CoW: 0.86 ± 0.04, sinuses: 0.85 ± 0.03. *p*-value for all vessels: ≥0.63. ICAD: CoW + sinuses: 0.81 ± 0.04. CoW: 0.84 ± 0.04. Sinuses: 0.79 ± 0.05. *p*-value for all vessels: ≥0.10).

**Table 7:** Performance results for the second observer.

Parameter/location	Observer 1 vs. Observer 2		Automated vs. Observer 2	
DS	Controls	ICAD	Controls	ICAD
CoW + sinus	**0.85** (0.81–0.90)	**0.80** (0.74–0.86)**	**0.86** (0.82–0.89)	**0.82** (0.78–0.86)**
CoW	**0.87** (0.80–0.91)	**0.83** (0.79–0.90)	**0.86** (0.81–0.99)	**0.83** (0.78–0.86)*
Sinus	**0.85** (0.78–0.91)	**0.80** (0.71–0.86)**	**0.86** (0.83–0.89)	**0.81** (0.74–0.88)*
HD [mm]	Controls	ICAD	Controls	ICAD
CoW + sinus	**8.7** (5.4–11.3)	**8.9** (5.4–16.0)	**7.6** (4.6–9.2)	**9.3** (6.2–17.0)**
CoW	**6.8** (5.0–9.5)	**8.2** (5.4–16.0)	**7.3** (4.6–9.2)	**8.0** (3.0–18.6)
Sinus	**6.0** (3.3–11.3)	**8.4** (2.4–14.5)	**4.7** (2.2–8.4)	**8.6** (3.7–17.0)**^†^
ASSD [mm]	Controls	ICAD	Controls	ICAD
CoW + sinus	**0.15** (0.11–0.21)	**0.22** (0.14–0.39)**	**0.14** (0.11–0.20)	**0.21** (0.15–0.27)**
CoW	**0.12** (0.07–0.21)	**0.21** (0.11–0.41)*	**0.13** (0.11–0.25)	**0.18** (0.11–0.30)
Sinus	**0.15** (0.13–0.30)	**0.25** (0.13–0.38)*	**0.14** (0.11–0.16)	**0.21** (0.13–0.29)***

Left: Using the Observer 1 segmentation as reference. Right: Using the automated segmentation as reference. Analysis of the statistical significance between the control and ICAD group.

All results are stated as median values (in bold) and range values (in brackets).

Statistical significance relative to the Automated vs. Observer 1 comparison.

* *p* < 0.05.

** *p* < 0.01.

*** *p* < 0.001.

^†^ *p* < 0.05.

The same applies for the automated segmentation vs. Observer 2 DS values (Controls: CoW + sinuses: 0.86 ± 0.02, CoW: 0.85 ± 0.03, sinuses: 0.86 ± 0.02. ICAD: CoW + sinuses: 0.82 ± 0.03, CoW: 0.83 ± 0.02, sinuses: 0.81 ± 0.04. *p*-value for all vessels: >0.05). When comparing the Dice scores of both cohorts, however, significantly lower DS values were observed in the ICAD group in the Observer 1 vs. Observer 2 comparison (CoW + sinuses: *p* < 0.01. Sinuses: *p* < 0.01) and in the automated segmentation vs. Observer 2 comparison (CoW + Sinuses: *p* < 0.01. CoW: *p* = 0.022. Sinuses: *p* = 0.045).

For the Observer 1 vs. Observer 2 HD values, no differences relative to the automated segmentation vs. Observer 1 comparison were noticeable in the control group (CoW + sinuses: 8.4 ± 2.0 mm. CoW: 7.0 ± 1.5 mm. Sinuses: 6.6 ± 3.2 mm. *p*-value for all vessels: ≥0.51) and the ICAD group (CoW + Sinuses: 9.8 ± 3.3 mm. CoW: 8.6 ± 3.0 mm. Sinuses: 7.7 ± 3.8 mm. *p*-value for all vessels: ≥0.14).

Similar HD values are also noticeable for the automated segmentation vs. Observer 2 comparison (Controls: CoW+ sinuses: 7.5 ± 1.3 mm, CoW: 7.2 ± 1.5 mm, sinuses: 5.0 ± 2.0 mm. ICAD: CoW + sinuses: 10.3 ± 3.9 mm, CoW: 8.0 ± 4.4 mm, sinuses: 8.6 ± 3.9 mm), with no differences compared to the Observer 1 vs. Observer 2 HD values (*p* ≥ 0.34). However, the Observer 2 vs. automated segmentation comparison of the ICAD group featured larger HD values in the sinuses compared to the automated segmentation vs. Observer 1 HD values (*p* = 0.047). Furthermore, the intergroup comparison revealed no differences for the Observer 1 vs. Observer 2 HD values (*p*-value for all vessels ≥0.14) but for the automated segmentation vs. Observer 2 HD values (CoW + sinuses: *p* < 0.01, sinuses: *p* < 0.01).

For the ASSD values, no differences between the Observer 1 vs. Observer 2 and the automated segmentation vs. Observer 1 comparison were detected (Controls: CoW + sinuses: 0.18 ± 0.05 mm. CoW: 0.18 ± 0.05 mm. Sinuses: 0.18 ± 0.05 mm. *p*-value for all vessels: ≥0.79. ICAD: CoW + Sinuses: 0.24 ± 0.08 mm. CoW: 0.22 ± 0.09 mm. Sinuses: 0.25 ± 0.08 mm. *p*-value for all vessels: ≥0.16).

Similar results were also observed for the automated segmentation vs. Observer 2 comparison (Controls: 0.15 ± 0.03 mm, CoW: 0.15 ± 0.05 mm, sinuses: 0.14 ± 0.02 mm. ICAD: CoW + sinuses: 0.21 ± 0.04 mm, CoW: 0.18 ± 0.06 mm, sinuses: 0.22 ± 0.06 mm. *p*-value for all vessels: *p* ≥ 0.10). However, in the Observer 1 vs. Observer 2 comparisons of the CoW, larger ASSD values are noticeable in the ICAD group relative to the control group (*p* = 0.022). Furthermore, in both comparisons presented in Table 7, larger sinusoidal ASSD values were observed (CoW + sinuses: *p* < 0.01, sinuses: *p* ≤ 0.045).

In the following, the flow metrics and cross-sectional area values determined with the Observer 2 segmentation were compared with the results from the original manual segmentation. Tables 4–6 display the bias, limits of agreement, the ICC values, and the *p*-values for the comparisons between the two human observers. The corresponding correlation and Bland-Altman plots can be found in the Supplementary Figures S4–S6 in the supplement. No significant differences were observed for the cross-sectional area values (*p*-value for all vessels: ≥0.19), the peak velocity values (*p*-value for all vessels: ≥0.64), and the flow rates (*p*-value for all vessels: ≥0.39). Furthermore, the fce analysis yielded no significant differences (*p*-value for both groups: ≥0.94). However, the comparison between the control and the ICAD groups revealed a significant difference for the cross-sectional areas in the large arteries (*p* = 0.016) but not for the small arteries (*p* = 0.068) and the sinuses (*p* = 0.75). In addition, significant differences were observed for the flow rates in the small (*p* = 0.011) and large arteries (*p* < 0.01). No significant differences were observed for the flow rates in the sinuses (*p* = 0.36) and for all peak velocity values (*p* ≥ 0.18).

The Observer 2 results were furthermore compared with the values obtained with the CNN segmentation. Similar to the comparison with the original human observer, no significant differences were observed for the cross-sectional area values (*p*-value for all vessels: ≥0.16), the peak velocity values (*p*-value for all vessels: ≥0.84), the flow rates (*p*-value for all vessels: ≥0.39), and the fce (*p*-value for both groups: ≥0.82).

### 3.4 Segmentation performance in stenosed vessels

**Figure 3:**
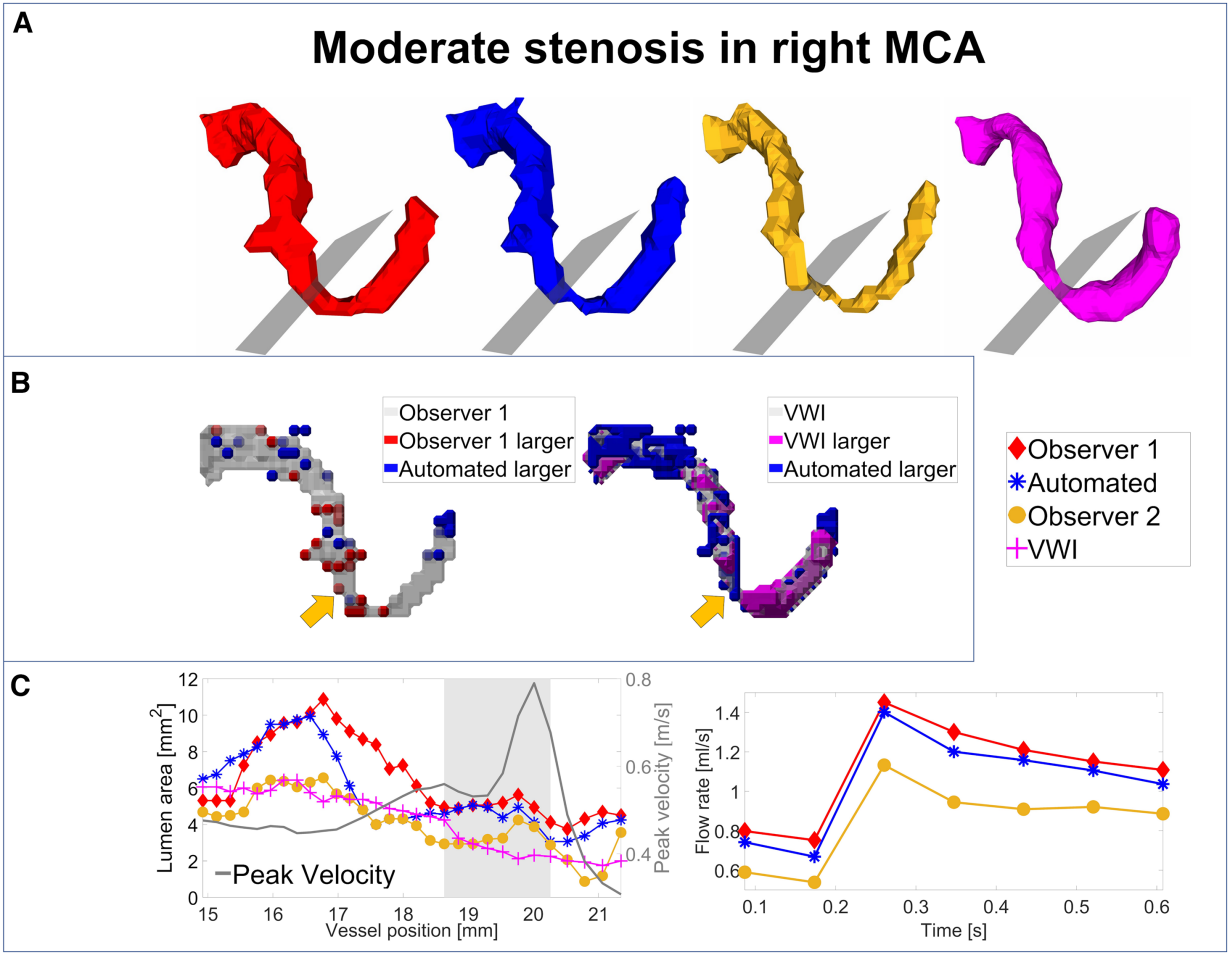
Analysis of segmentation performance in a moderate stenosis in the right MCA. (**A**) Isosurface renderings of Observer 1, Observer 2, automated and VWI segmentation. (**B**) Comparison between the automated and Observer 1 segmentation (left) and comparison between the automated and VWI segmentation (right). (**C**) Left: cross-sectional area and peak velocity profiles. Right: flow rates estimated in a region of interest around the stenosis (see grey shaded area in the profile plot) with the automated and Observer 1 and 2 segmentations. The stenosis is marked by analysis planes and orange arrows.

**Figure 4:**
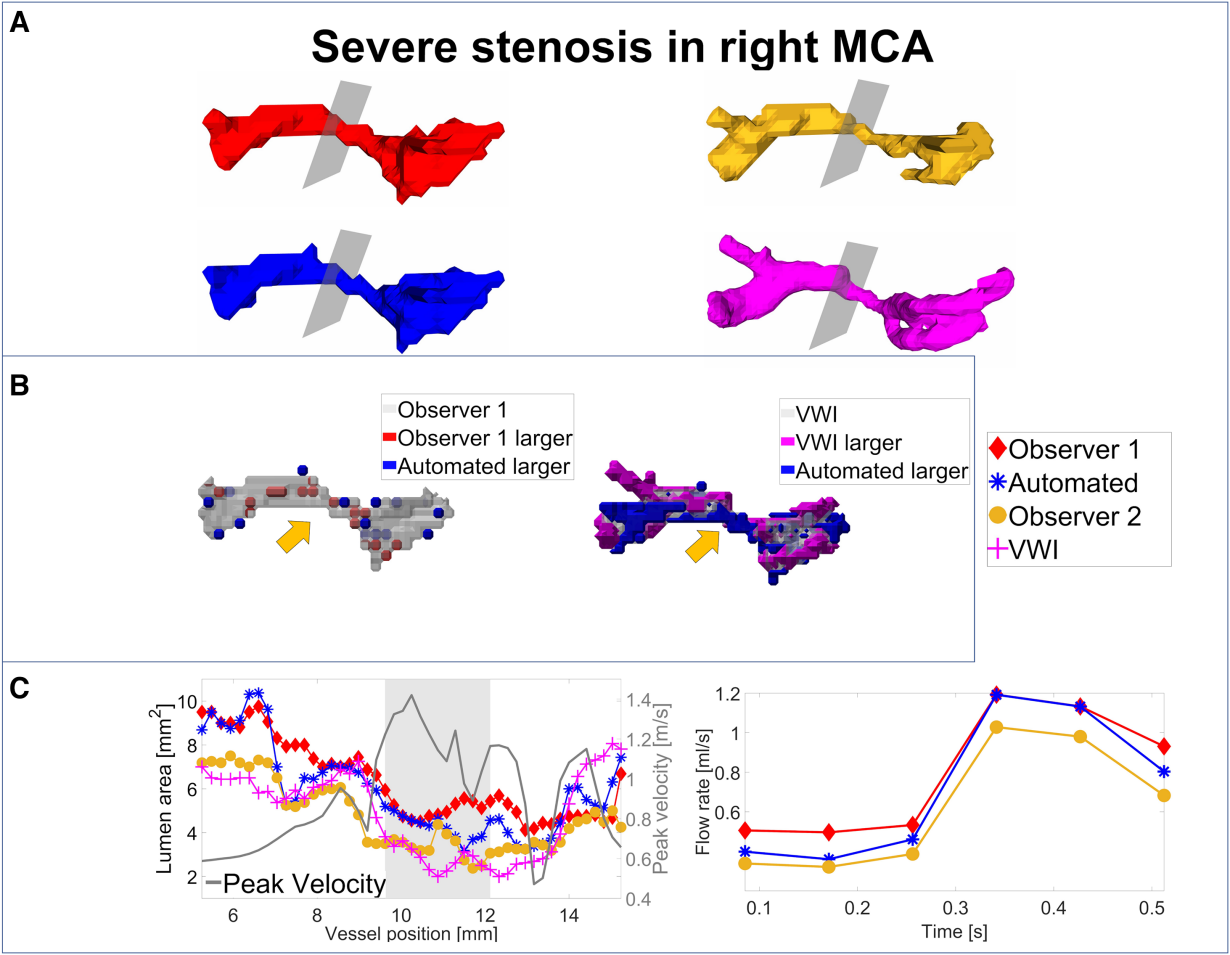
Analysis of segmentation performance in a severe stenosis in the right MCA. (**A**) Isosurface renderings of Observer 1, Observer 2, automated and VWI segmentation. (**B**) Comparison between the automated and Observer 1 segmentation (left) and comparison between the automated and VWI segmentation (right). (**C**) Left: cross-sectional area and peak velocity profiles. Right: flow rates estimated in a region of interest around the stenosis (see grey shaded area in the profile plot) with the automated and Observer 1 and 2 segmentations. The stenosis is marked by analysis planes and orange arrows.

**Figure 5:**
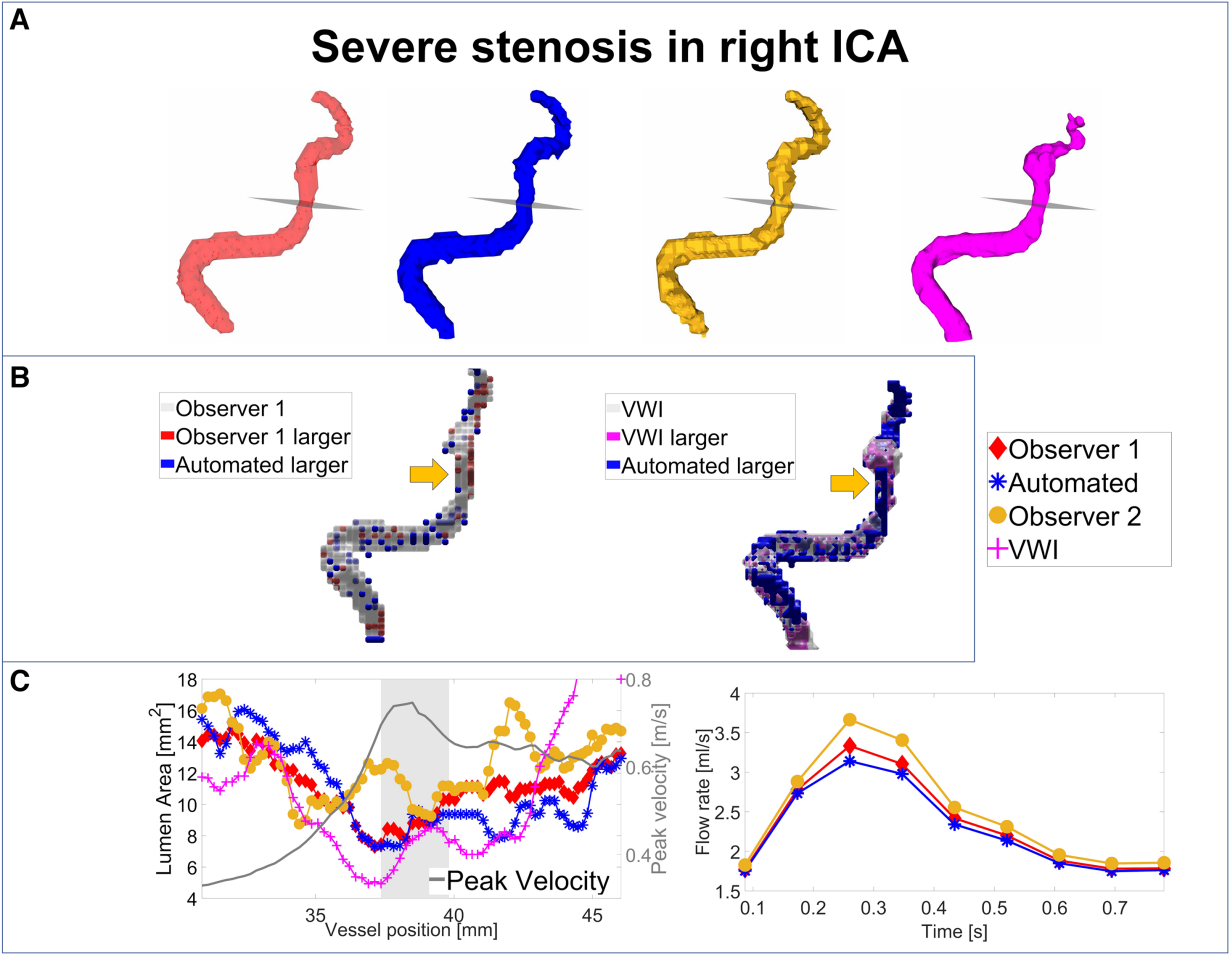
Analysis of segmentation performance in a severe stenosis in the right ICA. (**A**) Isosurface renderings of Observer 1, Observer 2, automated and VWI segmentation. (**B**) Comparison between the automated and Observer 1 segmentation (left) and comparison between the automated and VWI segmentation (right). (**C**) Left: cross-sectional area and peak velocity profiles. Right: flow rates estimated in a region of interest around the stenosis (see grey shaded area in the profile plot) with the automated and Observer 1 and 2 segmentations. The stenosis is marked by analysis planes and orange arrows.

For an analysis of the segmentation performance of the CNN in stenosed vessels, three intracranial stenosis cases were examined in further detail. Figures 3–5 display the segmentation results for a moderate (Figure 3) and a severe stenosis (Figure 4) in the right MCA and a severe stenosis in the right ICA (Figure 5). In Figures 3A, 4A, 5A the segmentation results of the stenosed vessel only are shown from Observer 1 (red), the automated segmentation (blue), Observer 2 (orange) and VWI (violet). Part B of the figures displays difference maps to illustrate the segmentation error between the automated segmentation and the Observer 1 (left) and between the automated segmentation and the VWI (right). The left side of part C of the figures shows profile plots of the lumen cross-sectional areas (for the Observer 1, automated, Observer 2 and VWI segmentations) as well as the peak velocity profiles. The gray shaded areas mark the location of the stenosis (see also the analysis planes in the segmentation plots in A and the orange arrow in the error maps in B). The plots on the right in part C of the figures show the time-resolved median flow rates around the stenosis (determined in the gray-shaded region above), assessed with the Observer 1 (red), the automated (blue), and the Observer 2 (orange) manual segmentation.

Table 8 illustrates the segmentation metrics of the stenosed vessels as well as statistical comparisons of the lumen area and flow rate values, determined around the stenosis (see gray shaded areas in the profile plots in Figures 3C–5C). Table 8A displays the DS, HD and ASSD values for the Observer 1 (O1) segmentation, the Observer 2 (O2) segmentation and the automated (CNN) segmentation using the VWI segmentation as reference. For calculation of segmentation metrics for this table, only the part of the vessel that was stenosed was considered. All three 4D flow MRI segmentations yield similar DS values (Observer 1: range 0.61–0.77. Observer 2: range 0.52–0.73. Automated segmentation: 0.59–0.75), similar HD values (Observer 1: range 2.1 mm–6.7 mm. Observer 2: range 2.3 mm–7.1 mm. Automated segmentation: range 2.6 mm–7.1 mm) and similar ASSD values (Observer 1: 0.20 mm–0.30 mm. Observer 2: 0.22 mm–0.45 mm. Automated segmentation: 0.23 mm–0.32 mm). Table 8B shows the lumen cross-sectional area values (median values and interquartile ranges) of the stenosed part of the vessels, estimated with the VWI segmentation, the Observer 1 (O1) segmentation, the Observer 2 (O2) segmentation and the automated (CNN) segmentation. In addition, bias and LOA values relative to the VWI segmentation are displayed. All three 4D flow segmentations feature a consistent overestimation relative to the segmentation obtained from the black blood images but good agreement (Observer 1: Overall bias ± LOA: 36.6 ± 16.1%, ICC: 0.93, *p* < 0.01. Observer 2: Overall bias ± LOA: 31.2 ± 42.4%, ICC: 0.84, *p* = 0.025. Automated segmentation: Overall bias ± LOA: 28.1 ± 13.9%, ICC: 0.93, *p* < 0.01). However, no differences were observed when comparing the automated segmentation with the manual segmentations (automated segmentation vs. Observer 1: Overall bias ± LOA: −8.8 ± 10.8%, ICC: 0.95, *p* = 0.05. Automated segmentation vs. Observer 2: Overall bias ± LOA: −3.2 ± 35.9%, ICC: 0.89, *p* = 0.15). Table 8C displays the flow rate values in the stenosed regions (median and interquartile ranges), estimated with both manual 4D flow segmentations and the automated segmentation. In addition, the table displays the bias and LOA values relative to the Observer 1 segmentation. No differences were observed when comparing the results from the automated segmentation with the manual segmentations of Observer 1 and 2 (automated segmentation vs. Observer 1: Overall bias ± LOA: −3.9 ± 4.3%, ICC > 0.99, *p* = 0.34. Automated segmentation vs. Observer 2: Overall bias ± LOA: −3.1 ± 13.9%, ICC: 0.98, *p* = 0.48).

**Table 8:** Analysis of the segmentation performance for three exemplary stenosed vessels.

Stenosis case	Example a Right MCA				Example b Right MCA				Example c Right ICA			
Stenosis grade [%]	>50				>70				>70			

A	Segmentation Metrics (Reference: VWI)			Segmentation Metrics (Reference: VWI)			Segmentation Metrics (Reference: VWI)		
Segmentation	O1	O2	CNN	O1	O2	CNN	O1	O2	CNN
DS	0.69	0.65	0.67	0.61	0.52	0.59	0.77	0.73	0.75
HD [mm]	2.1	2.3	2.6	3.1	4.3	4.0	6.7	7.1	7.1
ASSD [mm]	0.20	0.22	0.23	0.30	0.45	0.32	0.24	0.28	0.26

B	Lumen Area				Lumen Area				Lumen Area			
Segmentation	VWI	O1	O2	CNN	VWI	O1	O2	CNN	VWI	O1	O2	CNN
**Median** [mm^2^] (Iqr.) [mm^2^]	**2.59** (0.81)	**5.00** (0.22)	**3.06** (0.62)	**4.72** (0.69)	**2.88** (0.97)	**5.13** (0.69)	**3.31** (0.65)	**4.45** (0.84)	**7.7** (2.1)	**8.81** (0.84)	**11.2** (2.3)	**8.8** (1.9)
Bias [%]	–	56.3	16.2	46.8	–	56.8	15.1	41.4	–	19.4	40.5	15.9
LOA [%]	–	42.1	69.2	43.1	–	29.9	54.3	36.1	–	24.9	52.0	17.8

C	Flow rates			Flow rates			Flow rates		
Segmentation	O1	O2	CNN	O1	O2	CNN	O1	O2	CNN
**Median** (Iqr.) [ml/s]	**1.15** (0.31)	**0.89** (0.31)	**1.06** (0.31)	**0.77** (0.53)	**0.56** (0.65)	**0.64** (0.67)	**2.2** (1.1)	**2.3** (1.1)	**2.1** (1.0)
Bias [%]:	–	−24.3	−8.8	–	−26.3	−9.6	–	5.8	−2.3
LOA [%]	–	19.7	7.7	–	25.1	20.8	–	13.7	6.1

(**A**) Segmentation metrics (DS, HD and ASSD) of the stenosed vessels for the Observer 1 (O1), Observer 2 (O2) and automated (CNN) segmentation. The reference was the VWI segmentation. (**B**) Cross-sectional lumen area analysis for the VWI, Observer 1 and 2 and automated segmentation (median (bold) and interquartile range, Iqr.). Bias and LOA were calculated relative to the VWI segmentation. (**C**) Analysis of the flow rates (median (bold) and interquartile range, Iqr.). Bias and LOA were calculated relative to the Observer 1 segmentation.

## 4 Discussion

In this work, a CNN was trained using manually segmented intracranial 4D flow data and was successfully applied for the fully automated segmentation of stenosed intracranial vasculatures. In both a healthy control group and an ICAD patient cohort, similar segmentation performance could be achieved (comparison with Observer 1: median DS: ≥0.85 for controls, ≥0.84 for patients. Median HD: ≥6.3 mm for controls, ≥5.9 mm for patients. Median ASSD: ≥0.12 mm for controls, 0.21 mm for patients). In addition, no significant differences were observed when comparing the flow parameters and cross-sectional area values determined with the CNN segmentation with the original manual analysis and with the analysis performed by Observer 2. Interestingly, however, lower flow rates were observed in the CoW arteries in patients compared to controls regardless of the segmentation used for the analysis. These flow rate differences are likely due to the age difference of the two testing cohorts (see Table 1), as age related flow rate differences have already been reported by Wu et al. (27).

The automated segmentation required substantially less time than the manual segmentation performed by Observer 2 and was also significantly faster than reported for other automated segmentation techniques for intracranial 4D flow data (14). Furthermore, in contrast to manual segmentations, the CNN segmentation was not susceptible to often observed inter-observer variabilities (28).

Until recently, most deep learning-based segmentation networks of intracranial vasculature were based on TOF-MRA (29), CTA (30) or CTA in combination with DSA (31). Furthermore, the recent TopCoW challenge yielded very impressive results for CoW segmentations using CTA and MRA images (32). The large dataset available for this challenge may be used for transfer learning to further improve the segmentation performance of 4D flow images. A possible challenge, however, may be the large difference in voxel size between MRA [0.30 mm × 0.30 mm × 0.71 mm according to (32)] and 4D flow MRI (1 mm isotropic) and differences in image contrast, signal to noise ratio and artifacts due to the different sequence design. Furthermore, in contrast to the work presented in this paper, the TopCoW challenge only addressed the segmentation of the CoW arteries but not of veins such as the sinuses.

For 4D flow MRI, until recently, non-deep learning techniques such as threshold-based segmentation (33) or segmentation based on a centerline processing scheme (14) have been more common. Rothenberger et al. recently presented a post-processing technique using a standard difference of means, yielding average Dice score values of 0.76 for the CoW (15). However, in our study, we showed that using a deep learning approach, a larger mean Dice score of 0.85 could be achieved.

When comparing the Observer 2 segmentation with Observer 1 or the automated segmentation, no significant differences in segmentation performance were noticeable. Notable distinctions, however, were observed when comparing the control and stenosis cases. In both the Observer 1 vs. Observer 2 and the automated vs. Observer 2 comparisons, the ICAD group featured significantly lower Dice scores in the sinuses and overall larger ASSD values. Significantly larger ASSD values are also noticeable in the automated segmentations of the ICAD cases. One reason for the lower segmentation performance in the sinuses of the ICAD group may be the varying field of view size. In the control group, the number of slices varied between 40 and 44 while in the ICAD group, the number of slices was between 26 and 60. A too small FOV, however, may lead to incomplete coverage of the sinuses, which may exacerbate an accurate vessel segmentation. Variations in the number of slices may also partially explain the significantly larger ASSD values in the arteries, since a too small FOV may lead to insufficient coverage of the basilar artery and other vessels typically at the edges of the FOV such as the vertebral arteries. Another reason for the significantly larger ASSD values in the arteries may be the larger variability in vascular geometry noticeable in patients with ICAD due to the pathological changes caused by atherosclerosis and due to the significant differences in age between the two testing cohorts (see Table 1). Furthermore, especially in severely stenosed arteries, noticeable signal dropouts are often observed which aggravate accurate vessel segmentation.

The analysis of the cross-sectional area and flow metrics yielded no significant differences between the automated, the original manual, and the Observer 2 segmentation. In the large arteries, small bias and small limits of agreement are noticeable for both the control and the ICAD group. Slightly larger limits of agreement were observed in the small arteries, which may be attributed to partial volume effects due to the notably smaller vessel size. The reason for the larger variations observed in the sinuses may be explained again by the sometimes-incomplete coverage of the veins due to a too small FOV size. Also, exact segmentation of the sinuses is more challenging due to the much lower signal intensities as well as lower velocities in these vessels.

More detailed analyses of the segmentation performance in stenosed vessels revealed good performance of segmentation in stenosed areas that resulted in similar flow rates and cross-sectional area values between the automated segmentation and the two manual observers. Co-registration of 4D flow MRI with black blood vessel wall imaging confirmed correct segmentations of stenosed regions within the limits of spatial resolution of the 4D Flow MRI acquisition. As expected, an overestimation of the lumen areas relative to results obtained from black blood vessel wall imaging was noticeable in all 4D flow-based segmentations. Furthermore, due to the different sequence designs, the switching of the imaging gradients is different between the two imaging modalities. Thus, differences in image artifacts such as distortion, blurring and motion corruption are to be expected, exacerbating accurate co-registration. In this work, rigid co-registration was used to match the black blood segmentation with the 4D flow-based segmentations. For more accurate co-registration results, non-rigid co-registrations can be considered, however, this would increase the time investment of the post-processing and would be out of the scope of this study. In this work, we aimed to develop a CNN to automate intracranial vessel segmentation from 4D flow MRI data to ease the analysis of volumetric hemodynamic parameters. Our aim was not to use 4D flow MRI for the diagnosis of stenosis grade using luminal narrowing.

One limitation of this study is the small number of cases used for training and testing of the CNN architecture. In this work, 134 cases were used for training and 20 cases for testing. In contrast, 499 cases were used for training the CNN for automated aorta segmentation (16). The small number of training cases may be problematic since large variations in the CoW geometry have been reported (34). The limited number of training cases may also be a further explanation for the slightly worse segmentation performance of the sinuses in ICAD patients. Furthermore, the distribution of healthy and diseased training cases was skewed with 76 control cases but only 58 ICAD cases. However, we think that using as many training cases as possible was more important than an even distribution of healthy vs. ICAD training cases. In addition, due to the limited number of ICAD cases, we only had very few test cases. The random selection of the test cases caused an age-difference between the two testing cohorts, leading to significantly younger control cases compared to the ICAD patients (see Table 1). However, the focus of our study was to create a CNN for the segmentation of intracranial vessels in healthy controls as well as ICAD patients. This means that we selected as many as possible intracranial 4D flow MRI datasets while neglecting age matching. In a future more clinically focused study, quantitative results of the ICAD patients compared to an age- and gender matched healthy control cohort will be assessed. In addition, to further improve performance of the CNN, more stenosis cases as well as of other intracranial vascular diseases will be incorporated to further improve the segmentation performance and for a generalization of the automated segmentation.

## 5 Conclusion

In this work, a deep learning-based approach was presented for the fully automated vessel segmentation of intracranial 4D flow MRI data of healthy subjects and stenosis patients. The introduced CNN segmentation took only 2.2 s on average to complete. The automated segmentations of the intracranial arteries and veins are in very good agreement with the manual segmentations of two independent observers and the analysis of lumen cross-sectional areas and flow metrics yielded no significant differences between manual and automated segmentations. Furthermore, the accuracy of the automated segmentation of stenosed intracranial arteries could be verified by co-registered vessel wall imaging. The automation of intracranial vessel segmentation significantly reduces the analysis time and may improve the robustness of determining hemodynamic parameters with intracranial 4D flow MRI. This work could therefore be an integral factor in increasing its clinical application.

## Data Availability

The datasets presented in this article are not readily available because datasets from an IRB approved study involving patients were used. The source code for the convolutional neural network can be downloaded from https://github.com/drsol1986/AI-Segmentation-.git.

## Supplementary material

Supplementary Figure S1, Supplementary Figure S2, Supplementary Figure S3, Supplementary Figure S4, Supplementary Figure S5, Supplementary Figure S6, Correlation and Bland-Altman analysis of the cross-sectional areas in the large arteries (**A**), small arteries (**B**) and sinuses (**C**): Comparison between the manual ground truth and the automated segmentation. Correlation and Bland-Altman analysis of the peak velocity values in the large arteries (**A**), small arteries (**B**) and sinuses (**C**): Comparison between the manual ground truth and the automated segmentation. Correlation and Bland-Altman analysis of the flow rates in the large arteries (**A**), small arteries (**B**) and sinuses (**C**): Comparison between the manual ground truth and the automated segmentation. Correlation and Bland-Altman analysis of the cross-sectional areas in the large arteries (**A**), small arteries (**B**) and sinuses (**C**): Comparison between the manual ground truth and the Observer 2 segmentation. Correlation and Bland-Altman analysis of the peak velocity values in the large arteries (**A**), small arteries (**B**) and sinuses (**C**): Comparison between the manual ground truth and the Observer 2 segmentation. Correlation and Bland-Altman analysis of the flow rates in the large arteries (**A**), small arteries (**B**) and sinuses (**C**): Comparison between the manual ground truth and the Observer 2 segmentation.
