# Supplementary figures and images for: Automated intracranial vessel segmentation of 4D flow MRI data in patients with atherosclerotic stenosis using a convolutional neural network
Source: Front Radiol. 2024 Jun 4;4:1385424. doi: 10.3389/fradi.2024.1385424 (PMC11183785; doi:10.3389/fradi.2024.1385424)

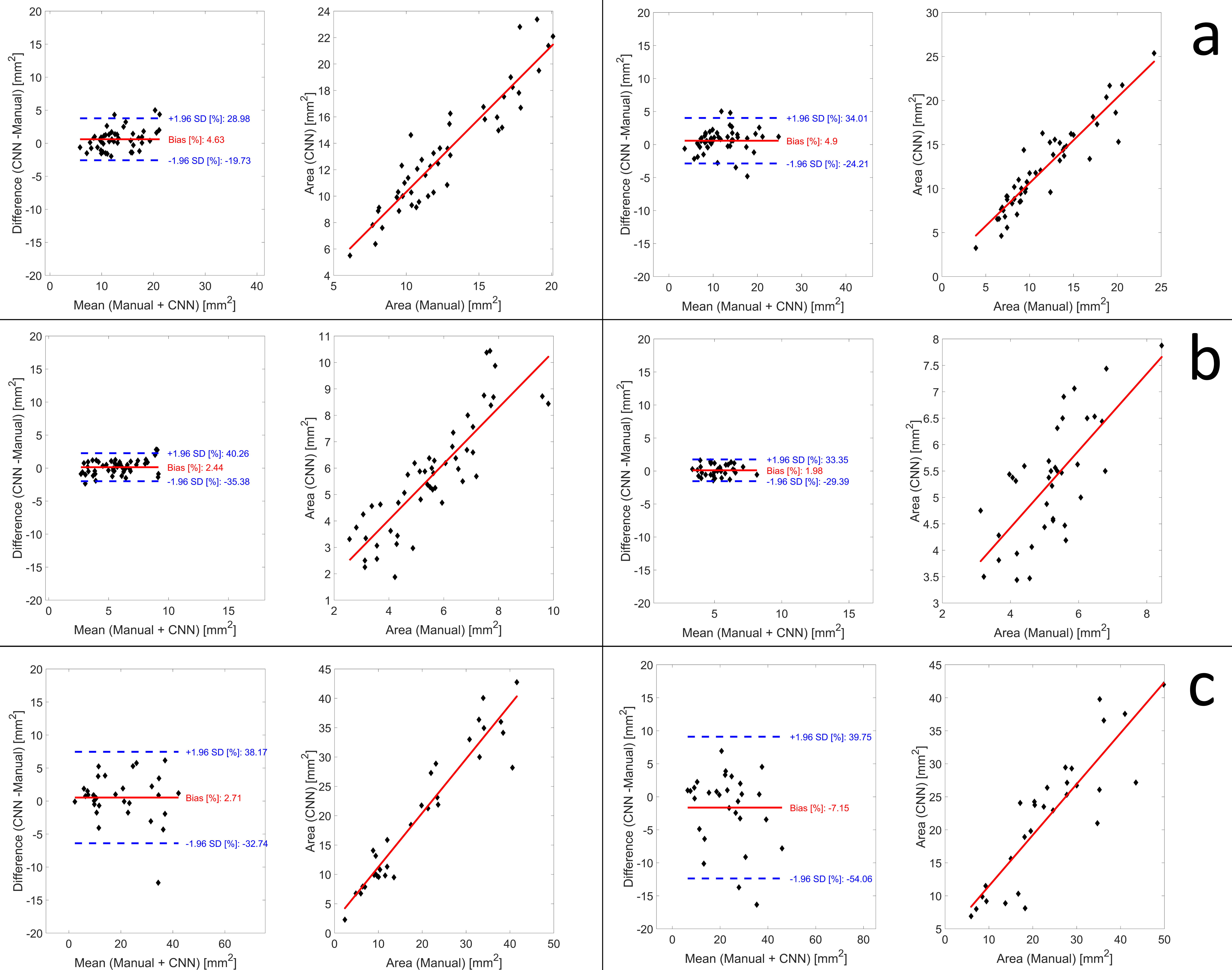

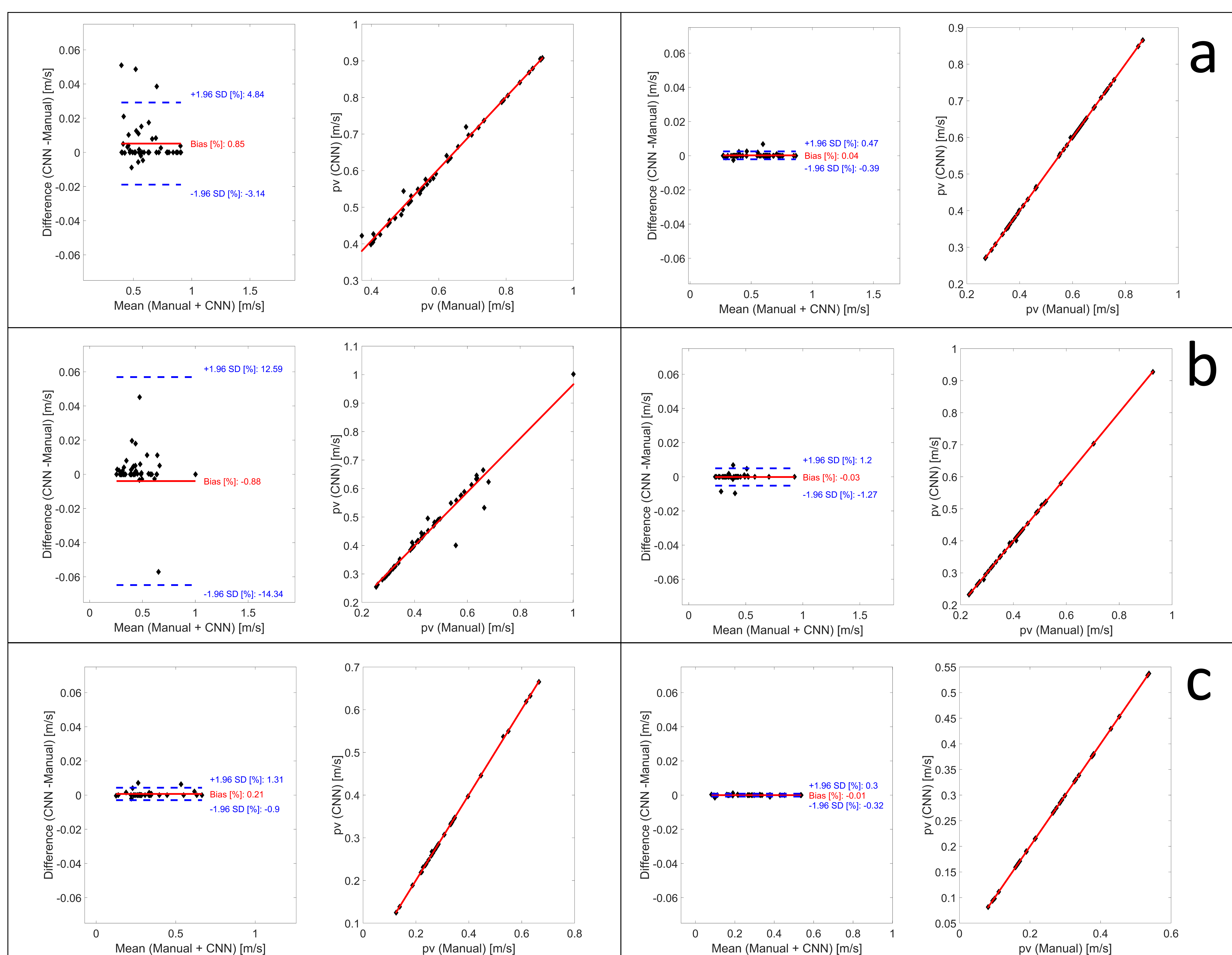

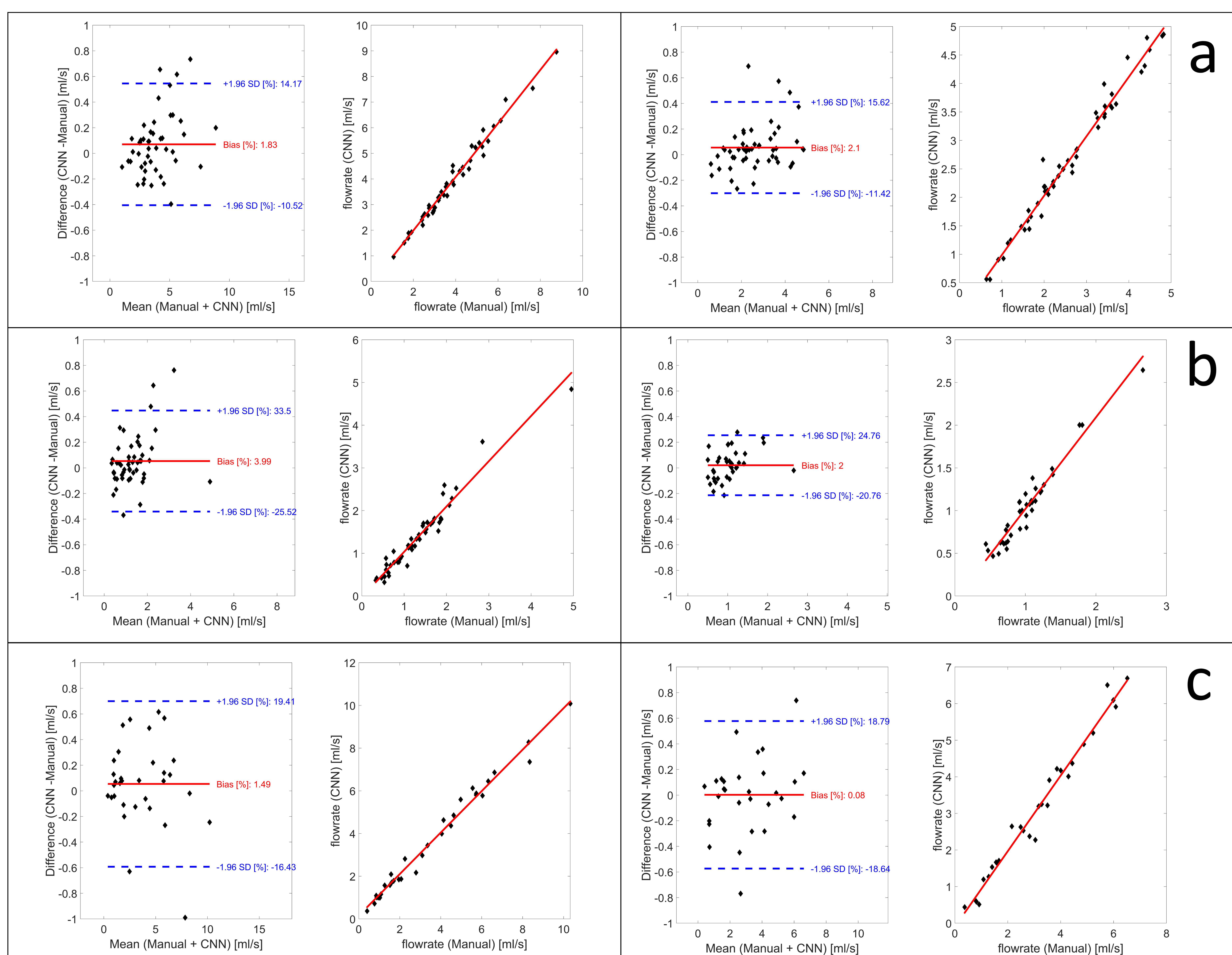

**Figure S3** Controls

**ICAD**

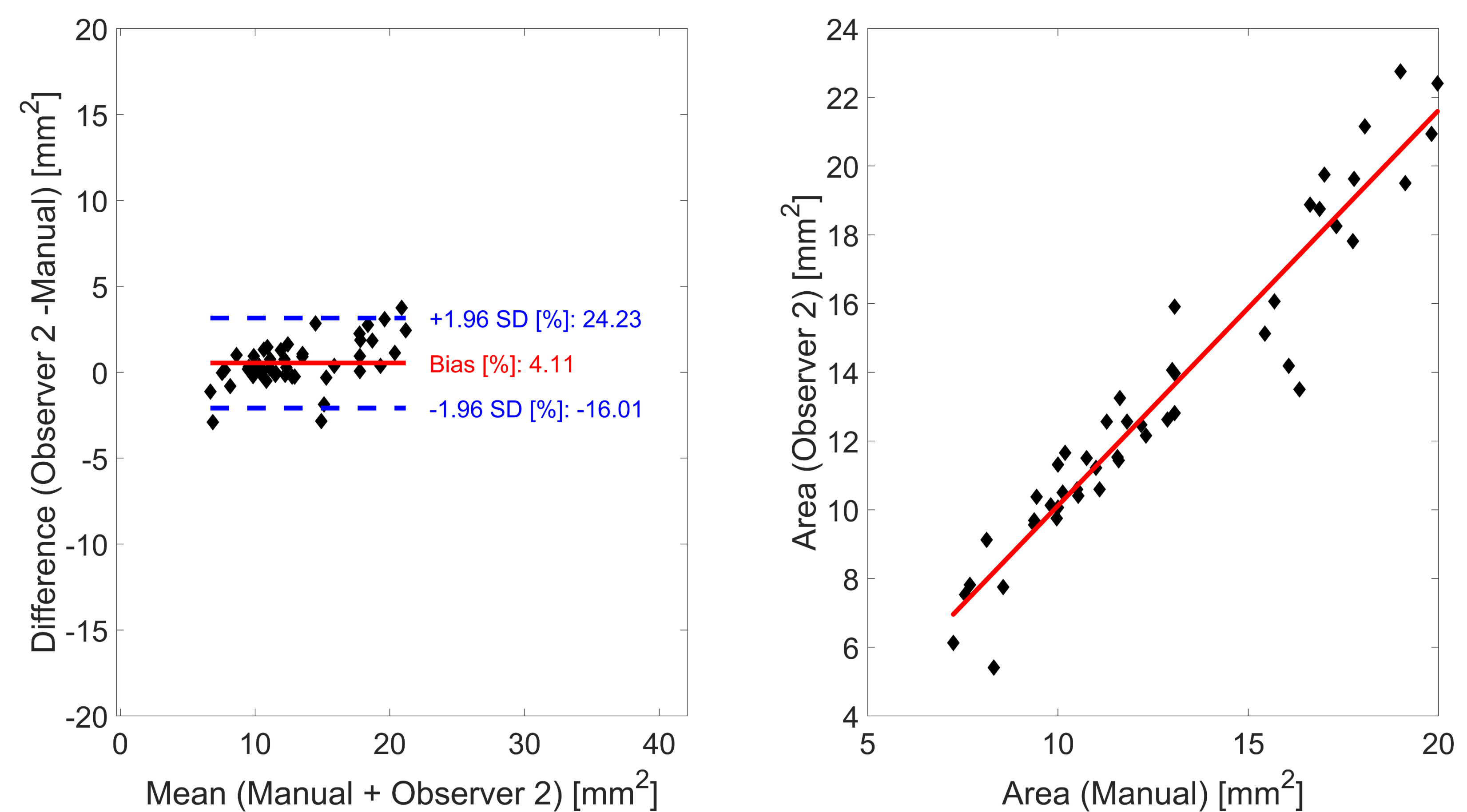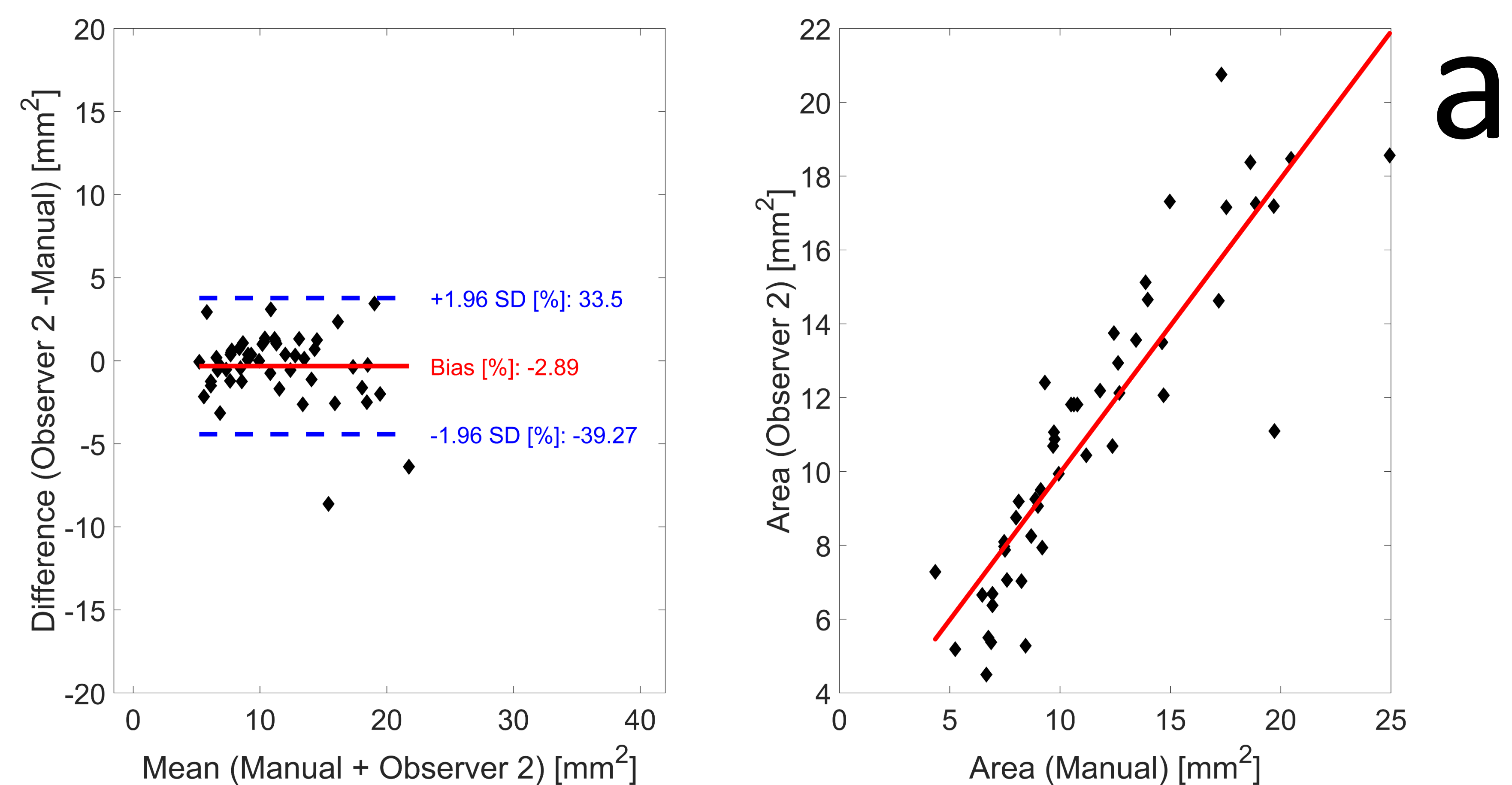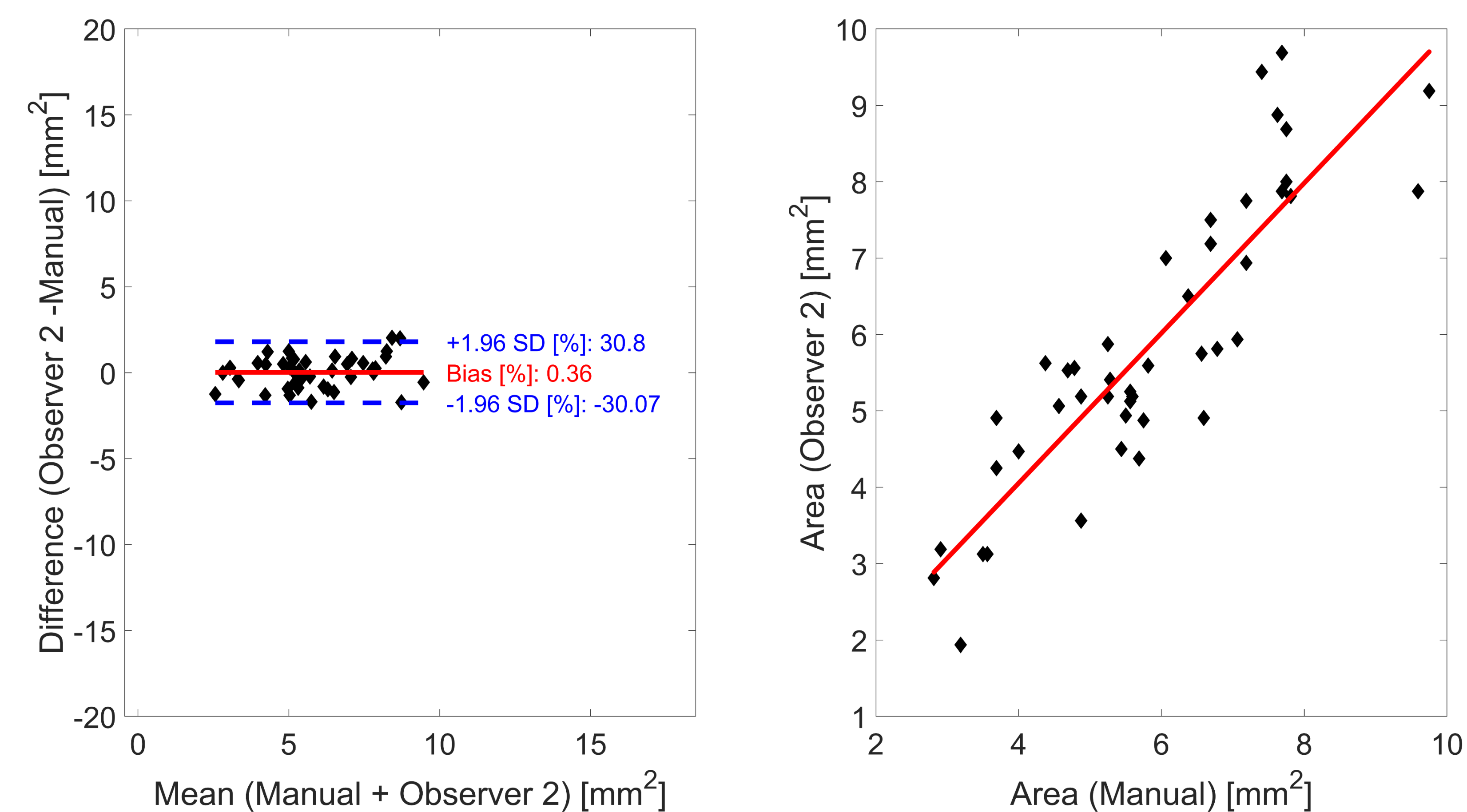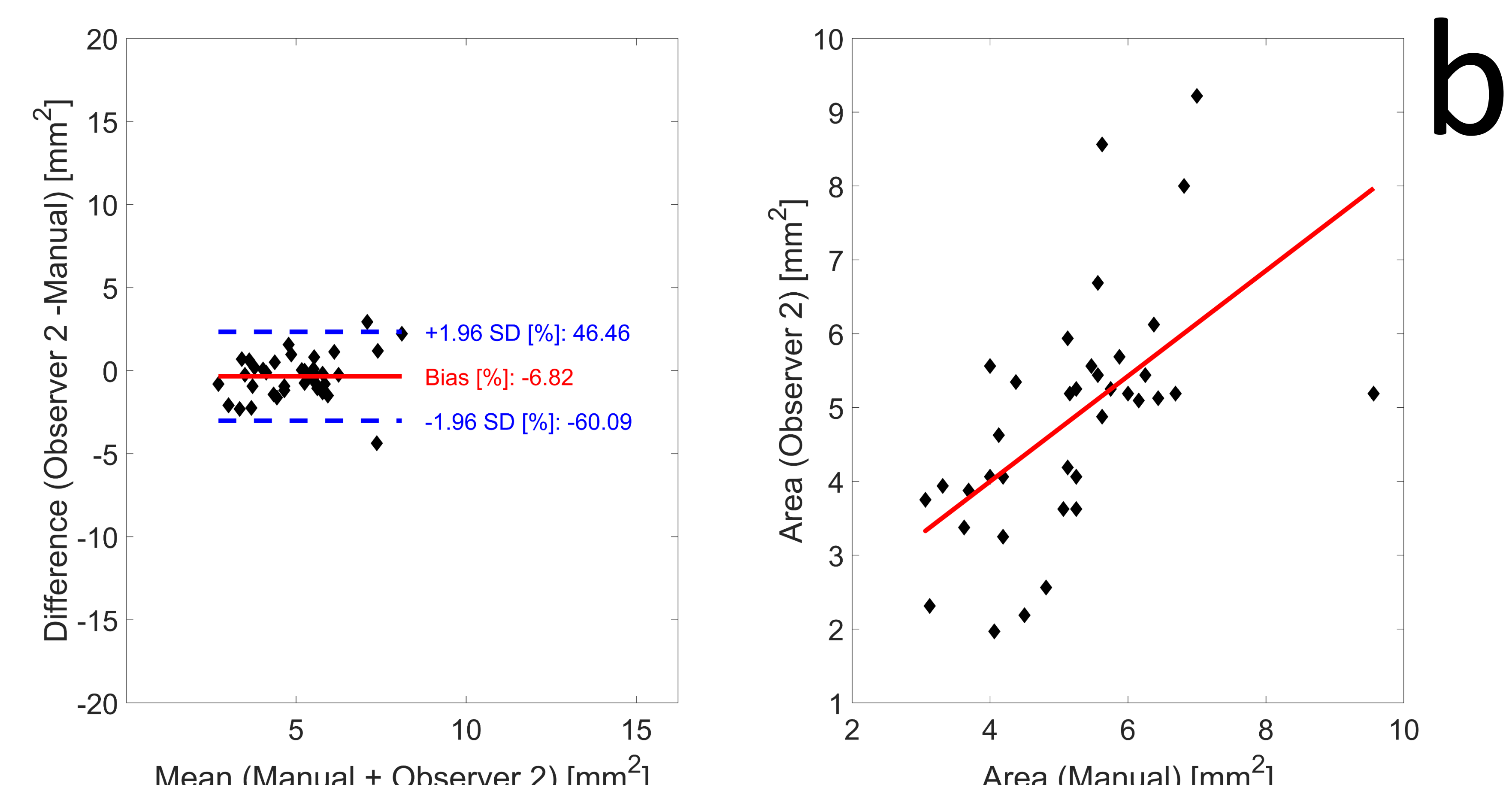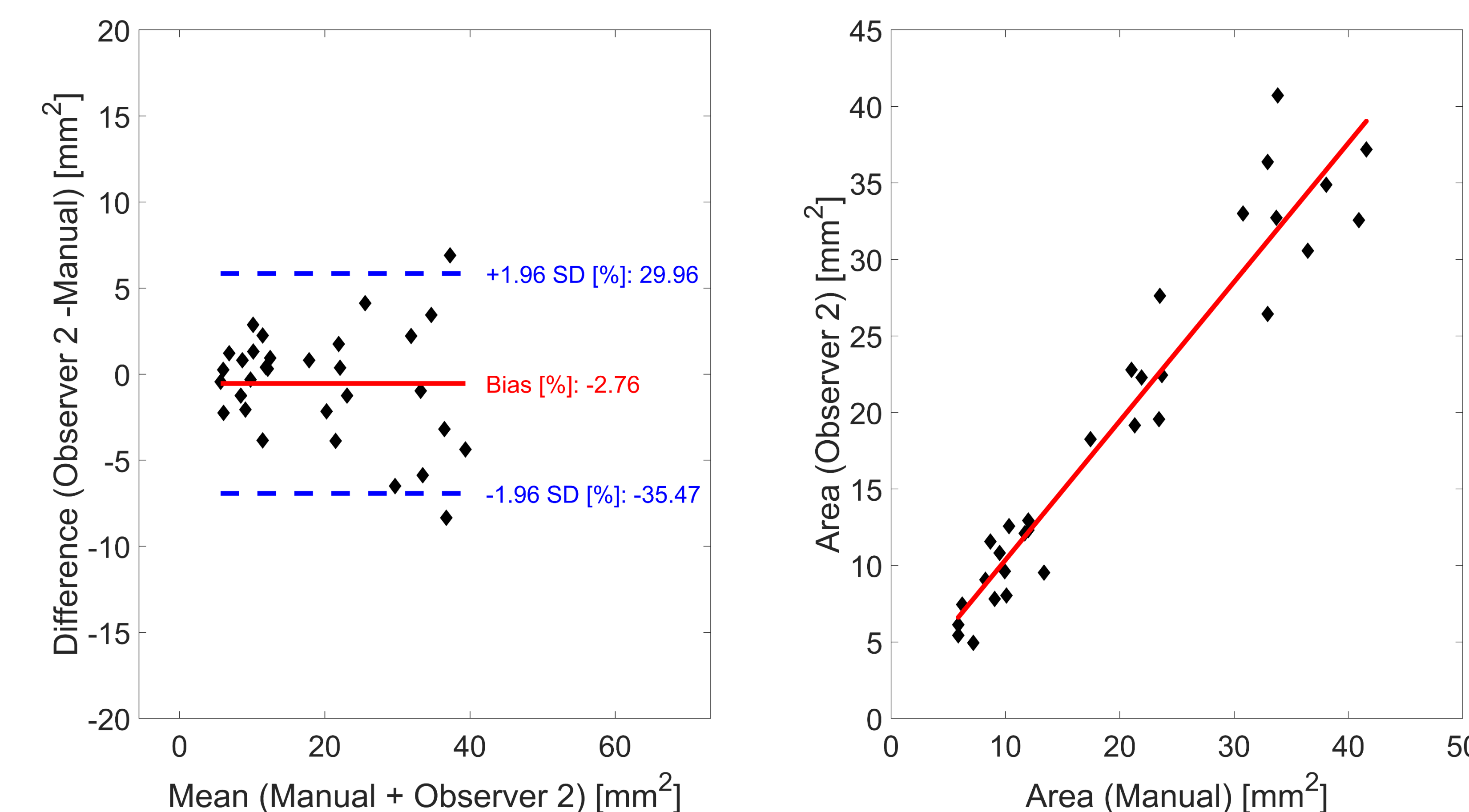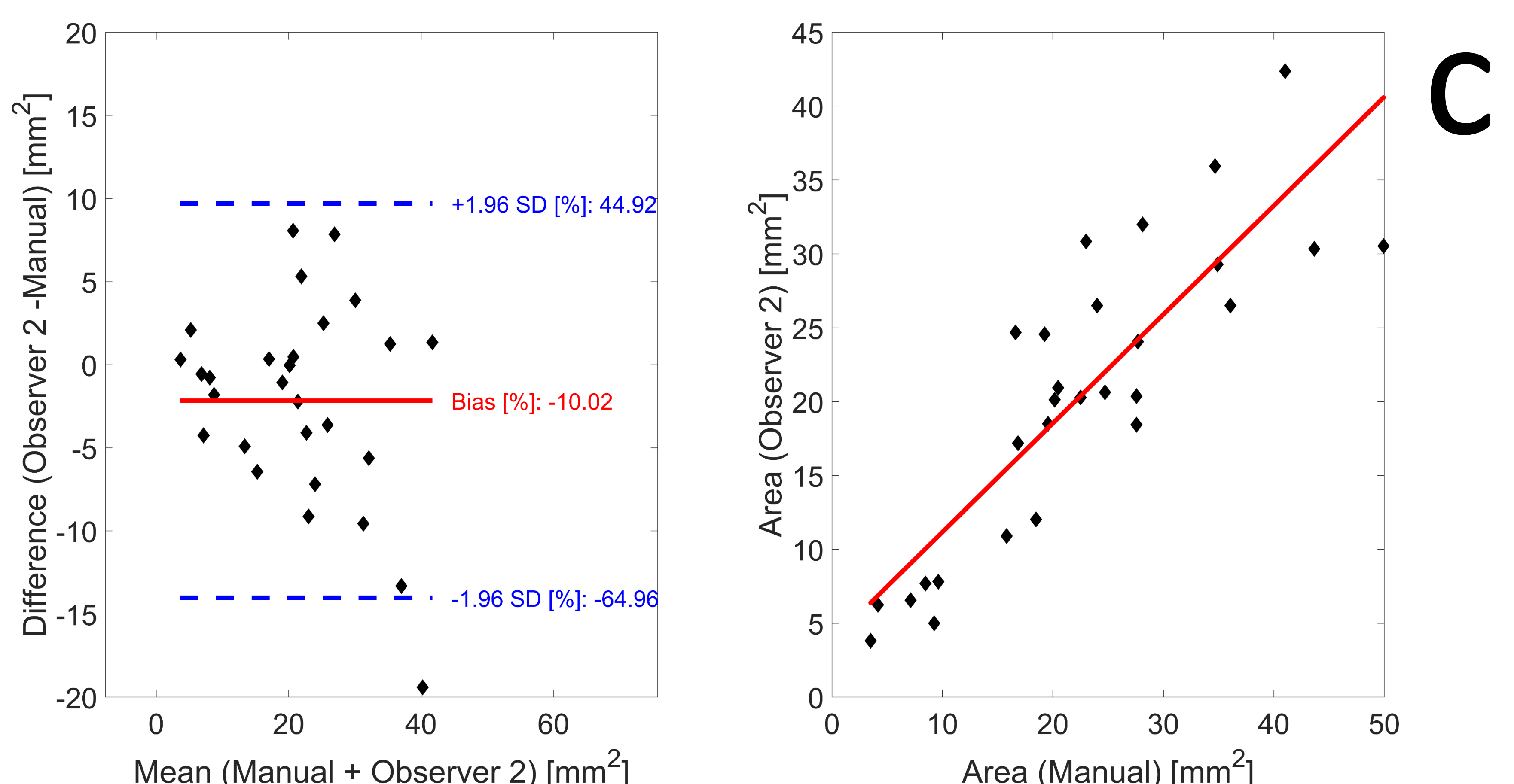

**Figure S4**

**Controls**

**ICAD**

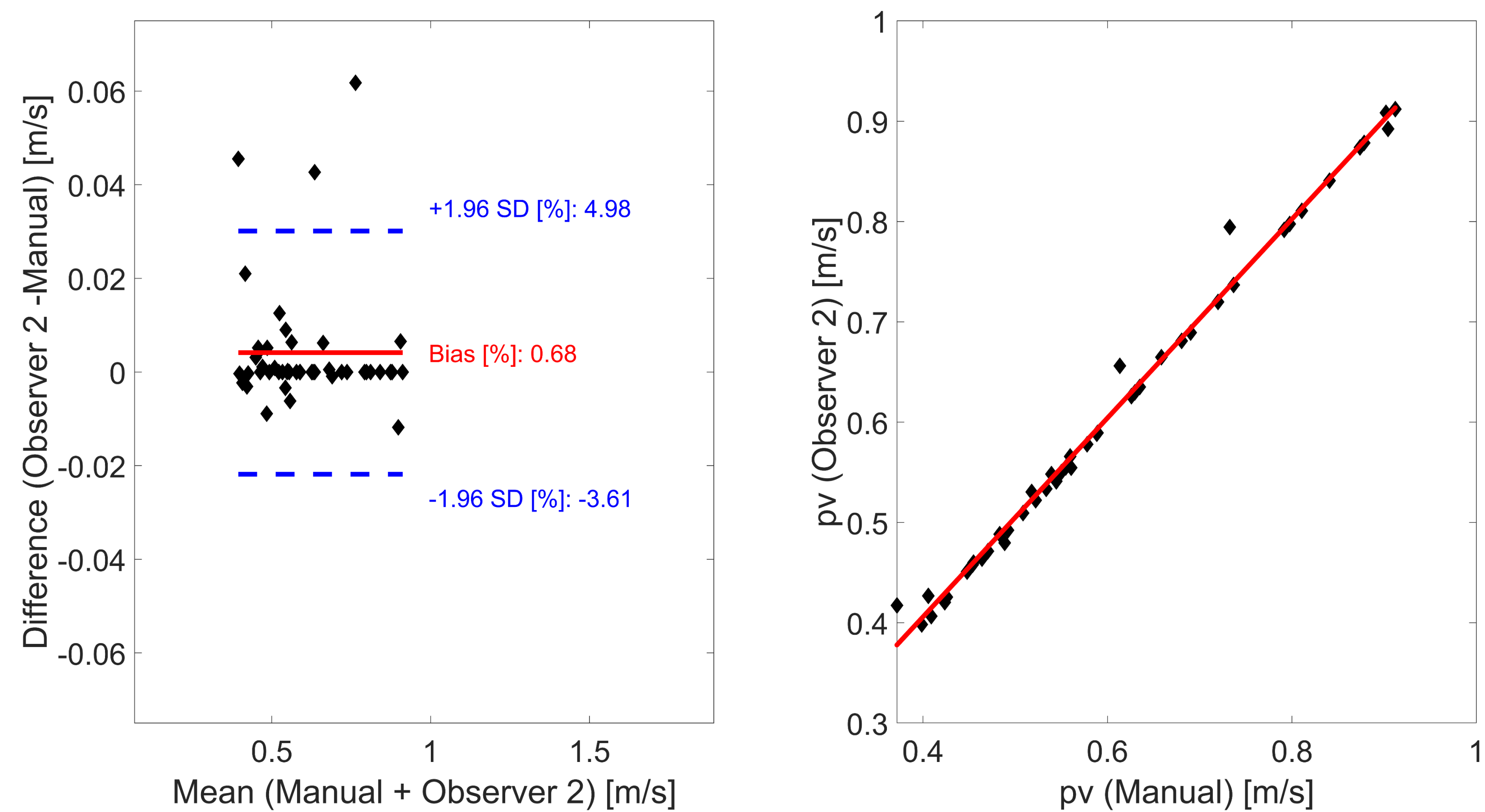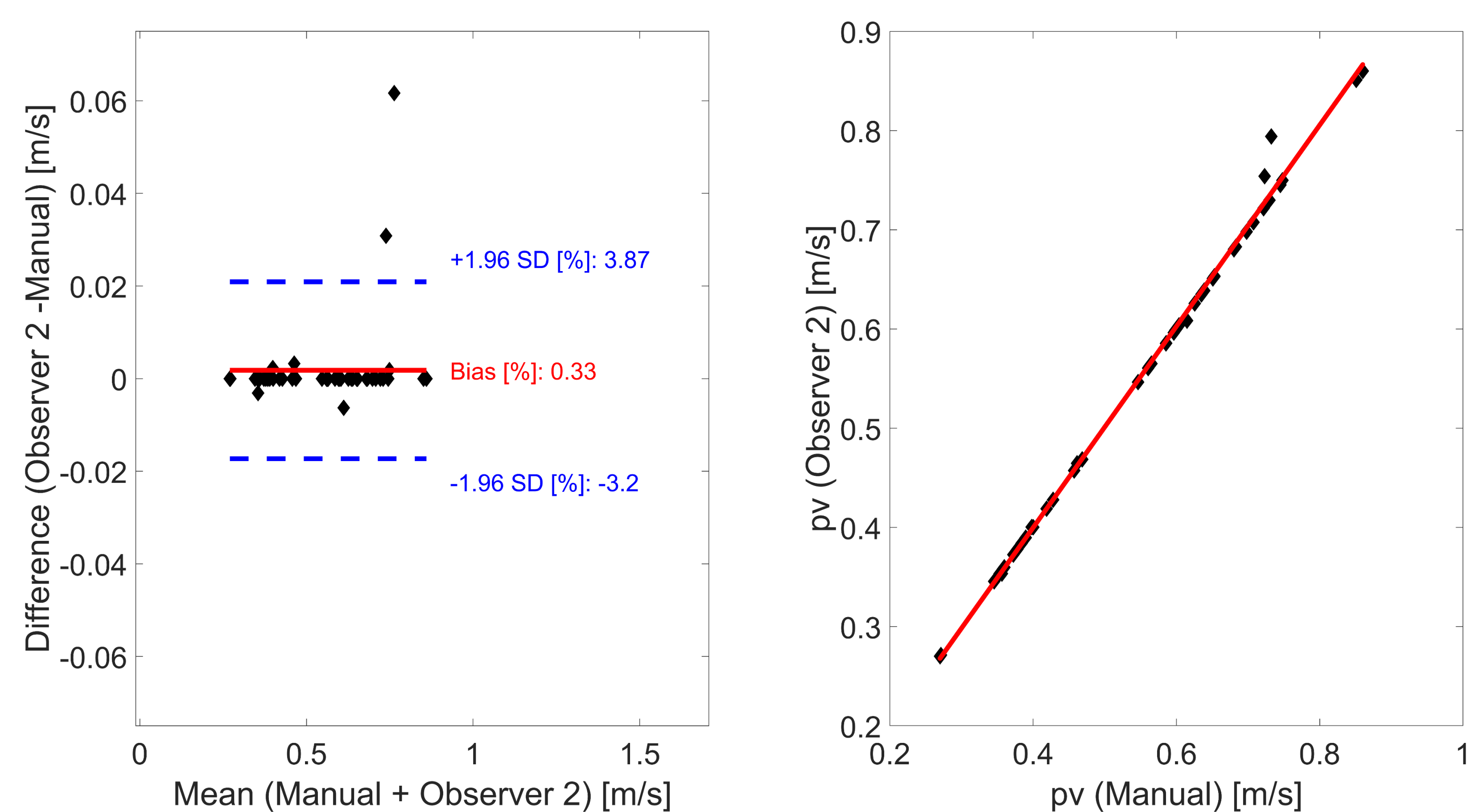

**a**

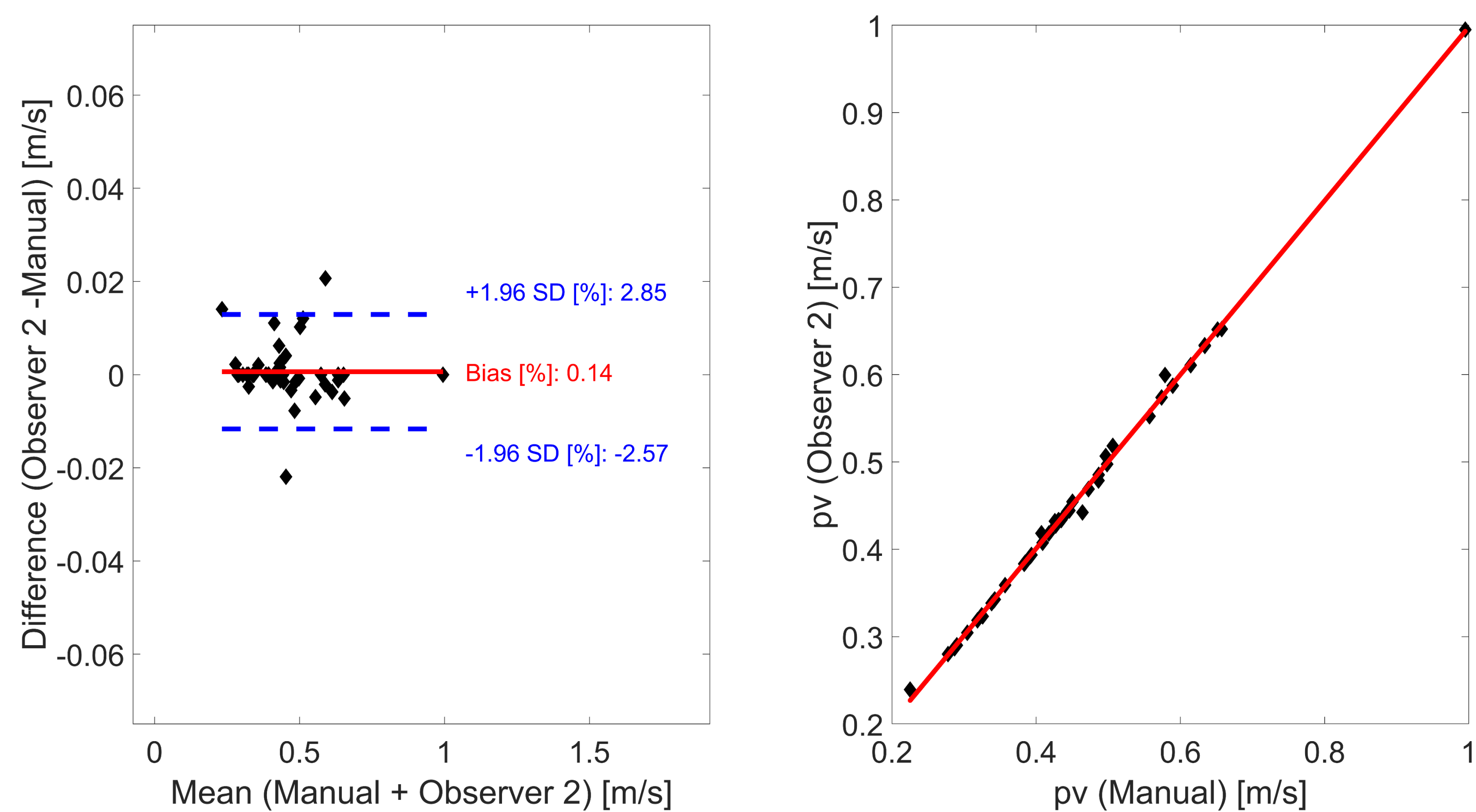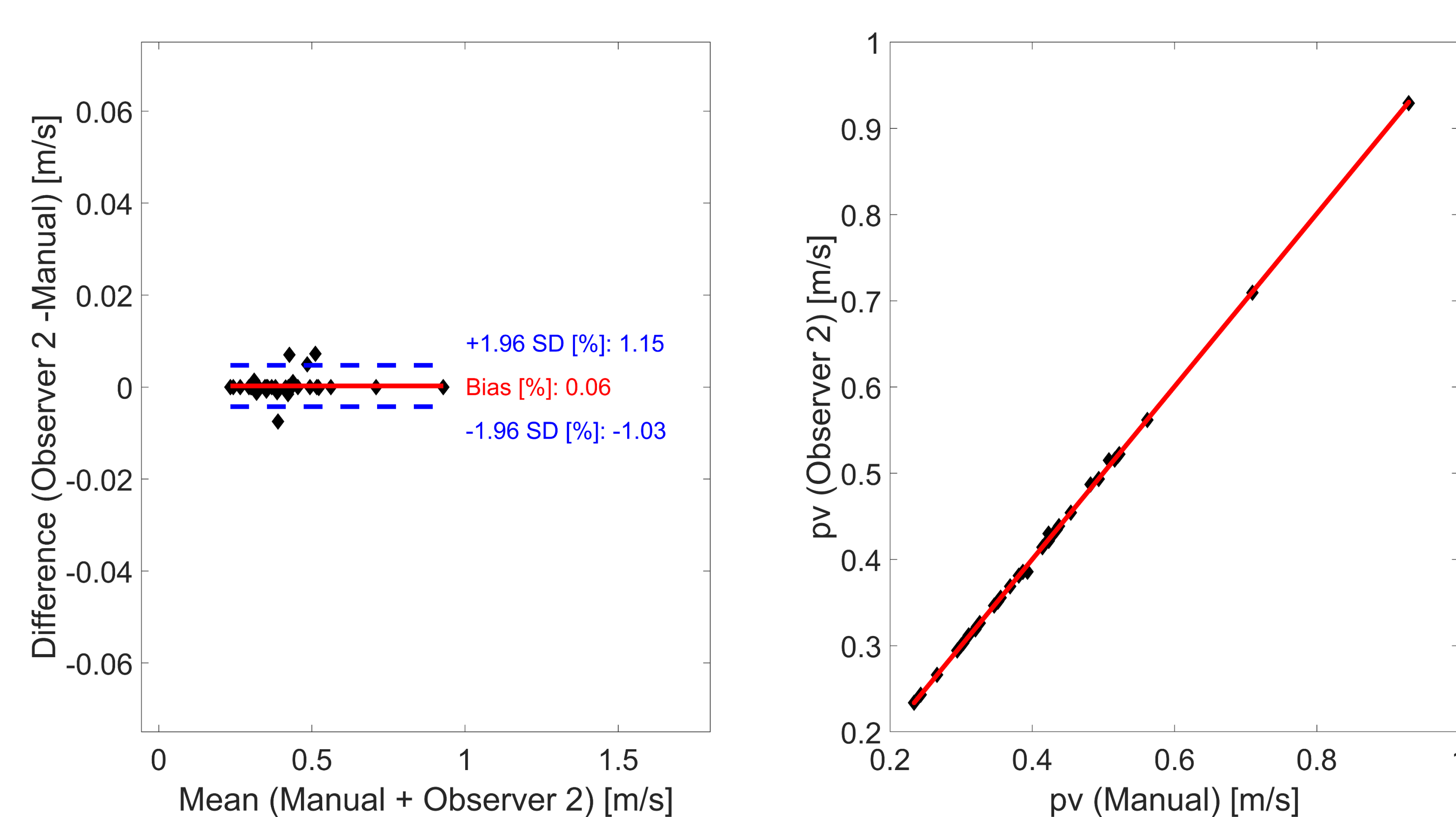

**b**

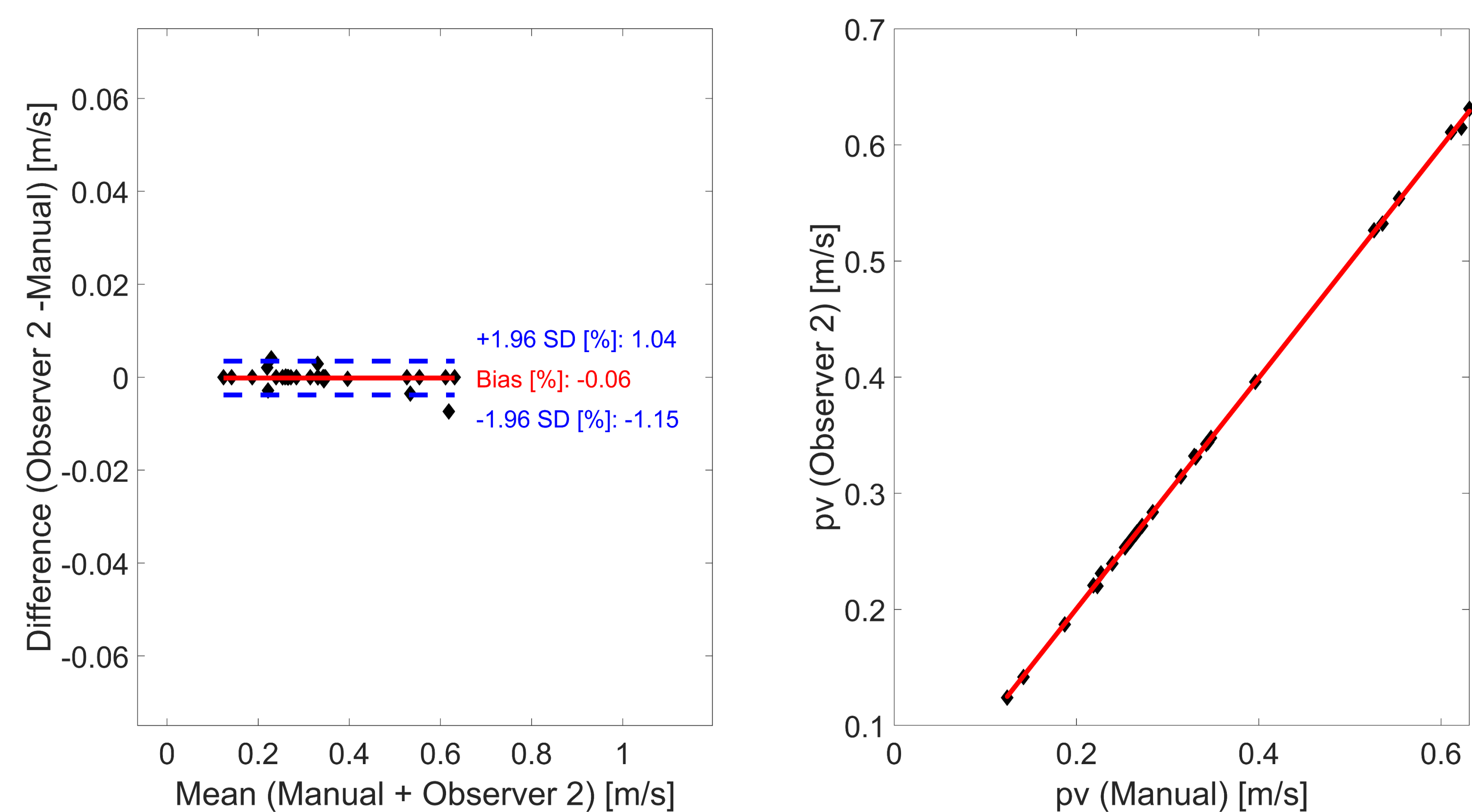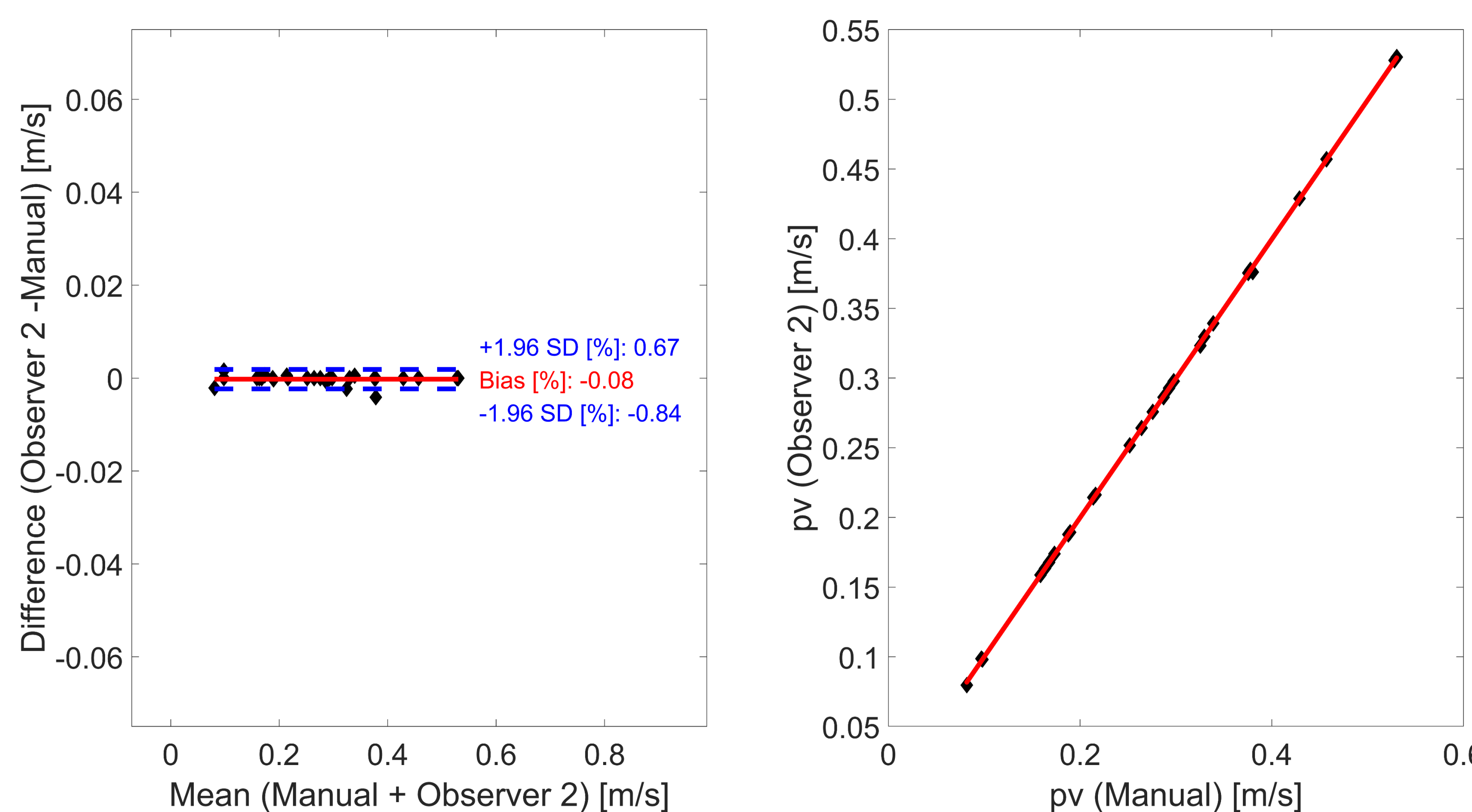

**c**

**Figure S5**

**Controls**

**ICAD**

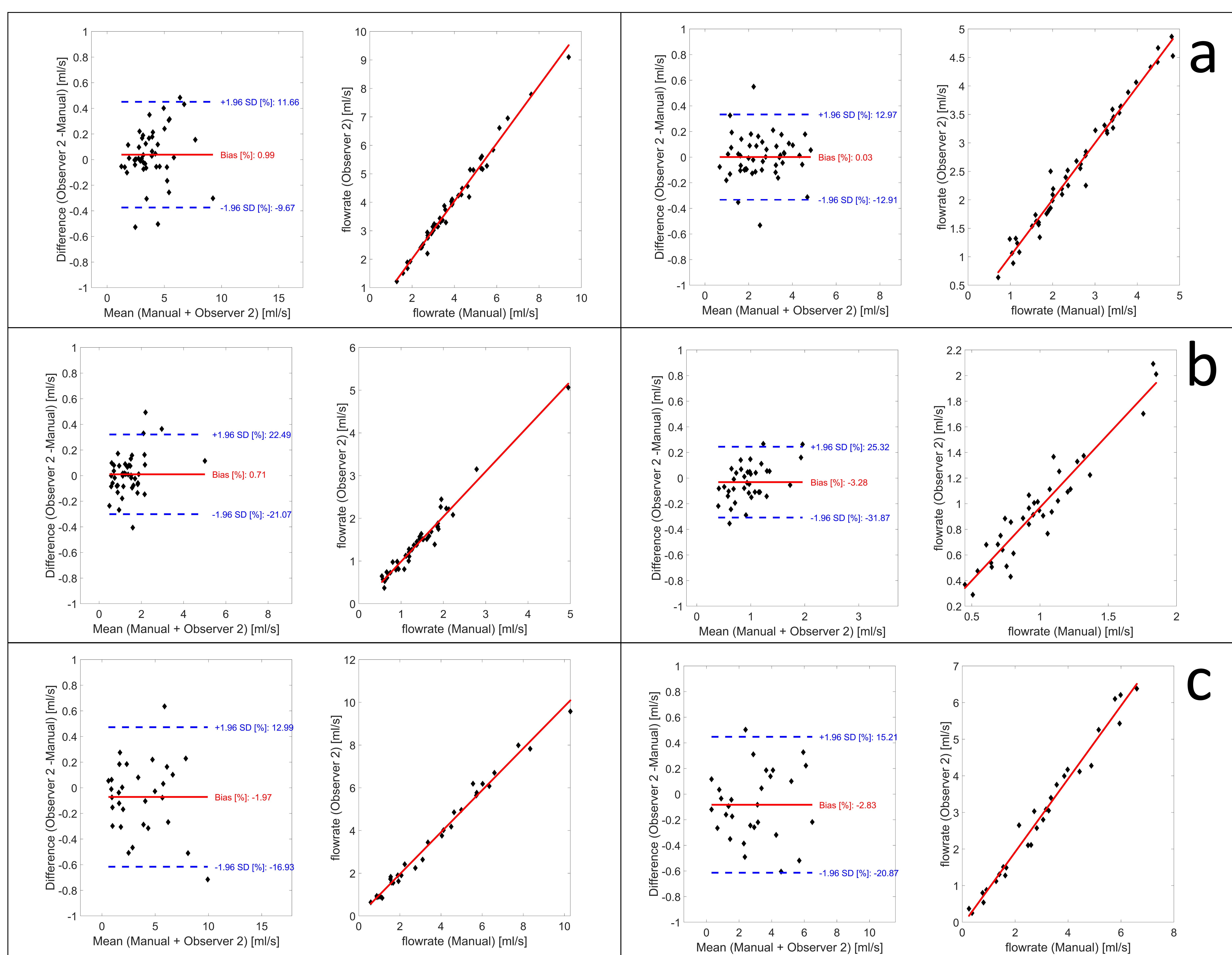

**Figure S6** Controls

ICAD

Supplement: Supplementary Figure S1, Supplementary Figure S2, Supplementary Figure S3, Supplementary Figure S4, Supplementary Figure S5, Supplementary Figure S6, — Correlation and Bland-Altman analysis of the cross-sectional areas in the large arteries (A), small arteries (B) and sinuses (C): Comparison between the manual ground truth and the automated segmentation. Correlation and Bland-Altman analysis of the peak velocity values in the large arteries (A), small arteries (B) and sinuses (C): Comparison between the manual ground truth and the automated segmentation. Correlation and Bland-Altman analysis of the flow rates in the large arteries (A), small arteries (B) and sinuses (C): Comparison between the manual ground truth and the automated segmentation. Correlation and Bland-Altman analysis of the cross-sectional areas in the large arteries (A), small arteries (B) and sinuses (C): Comparison between the manual ground truth and the Observer 2 segmentation. Correlation and Bland-Altman analysis of the peak velocity values in the large arteries (A), small arteries (B) and sinuses (C): Comparison between the manual ground truth and the Observer 2 segmentation. Correlation and Bland-Altman analysis of the flow rates in the large arteries (A), small arteries (B) and sinuses (C): Comparison between the manual ground truth and the Observer 2 segmentation. [file Datasheet1.pdf]
